# Design, synthesis, and biological evaluation of biotinylated colchicine derivatives as potential antitumor agents

**DOI:** 10.1080/14756366.2021.2013832

**Published:** 2021-12-16

**Authors:** Chao Wang, Yujing Zhang, Zeyu Wang, Yuelin Li, Qi Guan, Dongming Xing, Weige Zhang

**Affiliations:** a The Affiliated Hospital of Qingdao University, Cancer Institute, Qingdao University, Qingdao, Shandong, China; b The Affiliated Cardiovascular Hospital of Qingdao University, Qingdao University, Qingdao, Shandong, China; c Key Laboratory of Structure-Based Drug Design and Discovery, Ministry of Education, Shenyang Pharmaceutical University, Shenyang, Liaoning, China

**Keywords:** Colchicine, adverse effects, conjugate, biotin, disulphide bond

## Abstract

Chemical drug design based on the biochemical characteristics of cancer cells has become an important strategy for discovering new anti-tumour drugs to improve tumour targeting effects and reduce off-target toxicities. Colchicine is one of the most prominent and historically microtubule-targeting drugs, but its clinical applications are hindered by notorious adverse effects. In this study, we presented a novel tumour-specific conjugate **9** that consists of deacetylcolchicine (Deac), biotin, and a cleavable disulphide linker. **9** was found to exhibit potent anti-tumour activity and exerted higher selectivity between tumour and nontarget cells than Deac. The targeting moiety biotin might enhance the transport capability and selectivity of **9** to tumour cells via biotin receptor-mediated endocytosis. The tubulin polymerisation activity of **9** (with DTT) was close to the parent drug Deac. These preliminary results suggested that **9** is a high potency and reduced toxicity antitumor agent and worthy of further investigation.

## 1. Introduction

Chemotherapy is a mainstream anti-cancer treatment modality. However, its application is limited by its high toxicity and lack of specificity, which have serious adverse effects on surrounding non-cancerous cells and tissues^1^^,^^2^. In contrast, tumour-specific conjugates can be targeted accurately at the tumour sites under unique cancer microenvironment conditions such as low pH, high glutathione (GSH), various enzyme concentrations, or reactive oxygen species (ROS)^3–5^. This type of prodrug targeting can significantly improve the therapeutic index and reduce off-target toxicity^6^.

**Figure 1.**
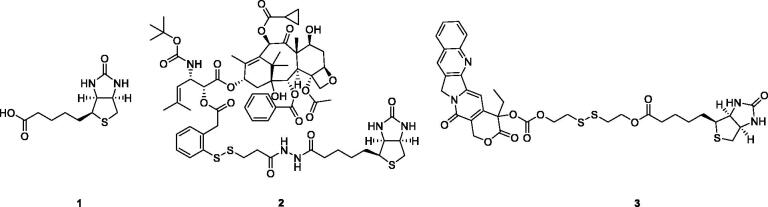
Chemical structures of biotin and biotin conjugates.

The biotin-receptor is a well-known tumour-associated receptor that is overexpressed in many cancers, including those of the breast, lung, ovarian, and renal^7^^,^^8^. Biotin-receptor binds biotin (**1**, Figure 1) with high affinity. Due to the overexpression of biotin-receptor uptake systems on the cell surface, biotin or biotin-conjugates can be recognised specifically and selectively by cancer cells, taken up preferentially by cancer cells. Biotin has caught a wide attention of many biologists and medical scientists due to its comparatively small molecular weight, high tumour-specificity, and simple biochemical structure. Various biotin-conjugates (e.g. **2** and **3**, Figure 1) have been developed and tested in culture cells and animal models with successful results^7^^,^^9^^,^^10^. Utilising biotin as a “tumor-targeting ligand” is a promising method for designing tumour-specific conjugates.

On discussion of the application of thiol in biological samples, GSH is always at the centre of attention because it is the most abundant thiol in cells. Given that cancer cells have much higher intracellular GSH concentration than normal cells and tissues, a variety of GSH-activatable conjugates (e.g. **2** and **3**) with disulphide bond linkage have been constructed with promising therapeutic efficacy^11^^,^^12^. A greater number of reports have shown that cellular GSH is indeed served as cellular redox switches involved in the bioreduction processes of redox-sensitive drugs^13–15^. Disulphide can be sheared along with the drug delivery system across a poorly reduced extra-cellular space to a strongly reduced intra-cellular microenvironment. This GSH-mediated prodrug strategy is one of the most promising drug delivery mechanisms proposed.

**Figure 2.**
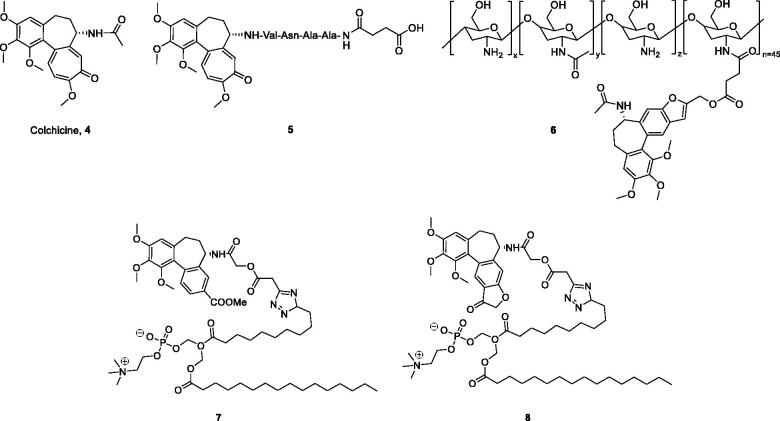
Chemical structures of colchicine and its derivatives.

Colchicine (**4**, Figure 2), an alkaloid extracted from plants of the genera Colchicum, Merendera, and Gloriosa, is one of the most prominent and historically microtubule-targeting drugs^16^^,^^17^. In the past several years, a wide variety of colchicine analogues have been reported^18–21^. Colchicine and its analogues possess the ability to bind irreversibly to tubulin, forming tubulin-colchicine complexes, which hinder microtubule formation and inhibit cell mitosis^22^. However, practical antitumor agents derived from colchicine have not been developed so far because of their low therapeutic indexes due to high toxicity effects in nontarget cells. The enhancement of therapeutic efficiencies and reduction of toxicity effects remain severe challenges for colchicine and its analogues’ application.

Recently, the efforts on the discovery of a series of novel colchicine prodrugs have been reported. In 2014, Robert Løvsletten Smith et al. have developed the legumain-cleavable peptide (Suc-Ala-Ala-Asn-Val)-colchicine (**5**, Figure 2). **5** was more toxic to cells over-expressing active legumain (M38L, HCT116) compared to cells expressing low levels of active or only prolegumain (HEK293, M4C, and SW620)^23^. In 2016, the chitosan-colchicinoid conjugate (**6**, Figure 2) was synthesised by fusing the succinic acid-modified furanoallocolchicine with polysaccharide chitosan^24^. **6** could induce *in vitro* tubulin reorganisation, cell cycle arrest, and inhibition of cell proliferation in 2D and 3D cultures. Furthermore, **6** demonstrated significantly better tumour growth inhibition than the parent drug possibly as a result of a better accumulation in the tumour. In 2019, prodrugs **7** and **8** (Figure 2) encapsulated into phosphatidylcholine-based enzyme responsive liposomes were reported by Ivan A. Boldyrev et al.^25^. Being exposed to elevated levels of phospholipases A2, especially in the case of a sPLA2 analogue, the liposomes release colchicinoid-containing fatty acids. The latter undergo further hydrolysis by non-specific esterases to release the active colchicinoid species. Noteworthy, **7** and **8** exhibited outstanding antiproliferative activity in the low nanomolar concentration range.

**Figure 3.**
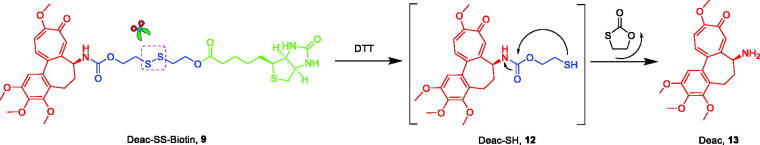
Reduction-sensitive drug release mechanism of Deac-SS-Biotin triggered by DTT (Glutathione mimetic).

The prodrug strategy is a practical approach in development of novel antitumor agents. Based on the above considerations, Deac-SS-Biotin (**9**, Figure 3) was developed by conjugating biotin with deacetylcolchicine (Deac), using a reduction-sensitive disulphide bond as a linkage to facilitate a rapid release of Deac in tumour cells. Some non-sensitive conjugates (**10** and **11**) were synthesised as the counterparts of Deac-SS-Biotin. In addition, the physiological stability, *in vitro* drug release, antiproliferative activity, and tubulin polymerisation activity of Deac-SS-Biotin were studied.

## 2. Result and discussion

### 2.1. Chemistry

#### 2.1.1. Synthesis of biotin conjugates

**Scheme 1.**
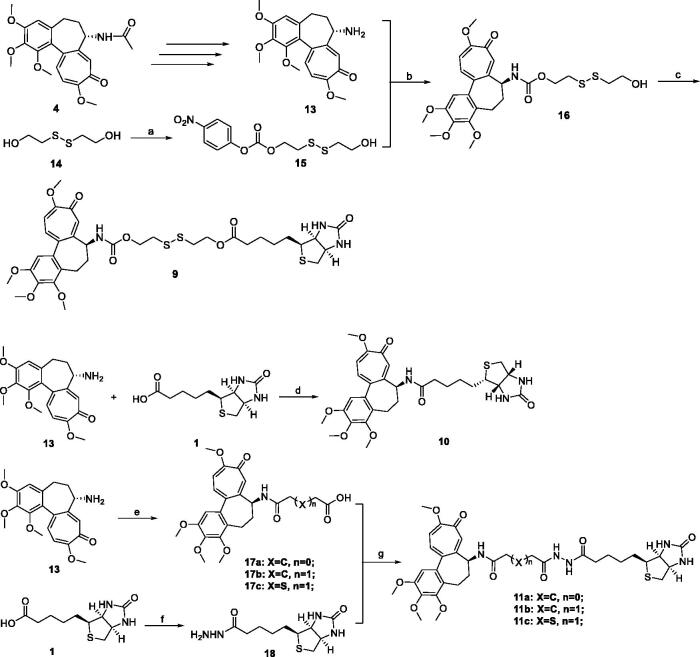
Reagents and conditions: (a) 4-nitrophenyl carbonochloridate, Et_3_N, THF, rt., 6 h; (b) THF, rt., 4 h; (c) **1**, DCC, DMAP, rt., 20 h; (d) HATU, Et_3_N, rt., 8 h; (e) anhydrides, NMM, DMSO, rt., 45 min; (f) (i) SOCl_2_, MeOH, rt., overnight; (ii) NH_2_NH_2_, rt., 17 h; (g) EDCI, HOBt, DMAP, Et_3_N, rt., 10 h.

The synthetic route for biotin-conjugates is depicted in Scheme 1. According to Bagnato’s method, Deac was synthesised in three steps^26^. The key intermediates **15**, **16**, **17**, and **18** were synthesised according to the established procedures^9^^,^^13^^,^^27^^,^^28^. At first, commercially available 2,2′-disulfanediyldiethanol (**14**) was treated with 4-nitrophenyl carbonochloridate in THF containing Et_3_N to obtain **15**, and then **15** was reacted with Deac to generate key intermediate **16**. Finally, the intermediate **16** was coupled with biotin to obtain Deac-SS-Biotin in the presence of DCC and DMAP. Furthermore, Deac-Biotin was obtained from the reaction of Deac and biotin in the presence of HATU and TEA. In addition, desacetylcolchicine derivatives **17** were prepared from Deac as reported by means of treatment with corresponding anhydrides, and then **17** was coupled with biotin hydrazine (**18**) in the presence of EDCI, HOBt, and DMAP to obtain Deac-CC-Biotins (**11**).

#### 2.1.2. Physiological stability of Deac-SS-Biotin

**Figure 4.**
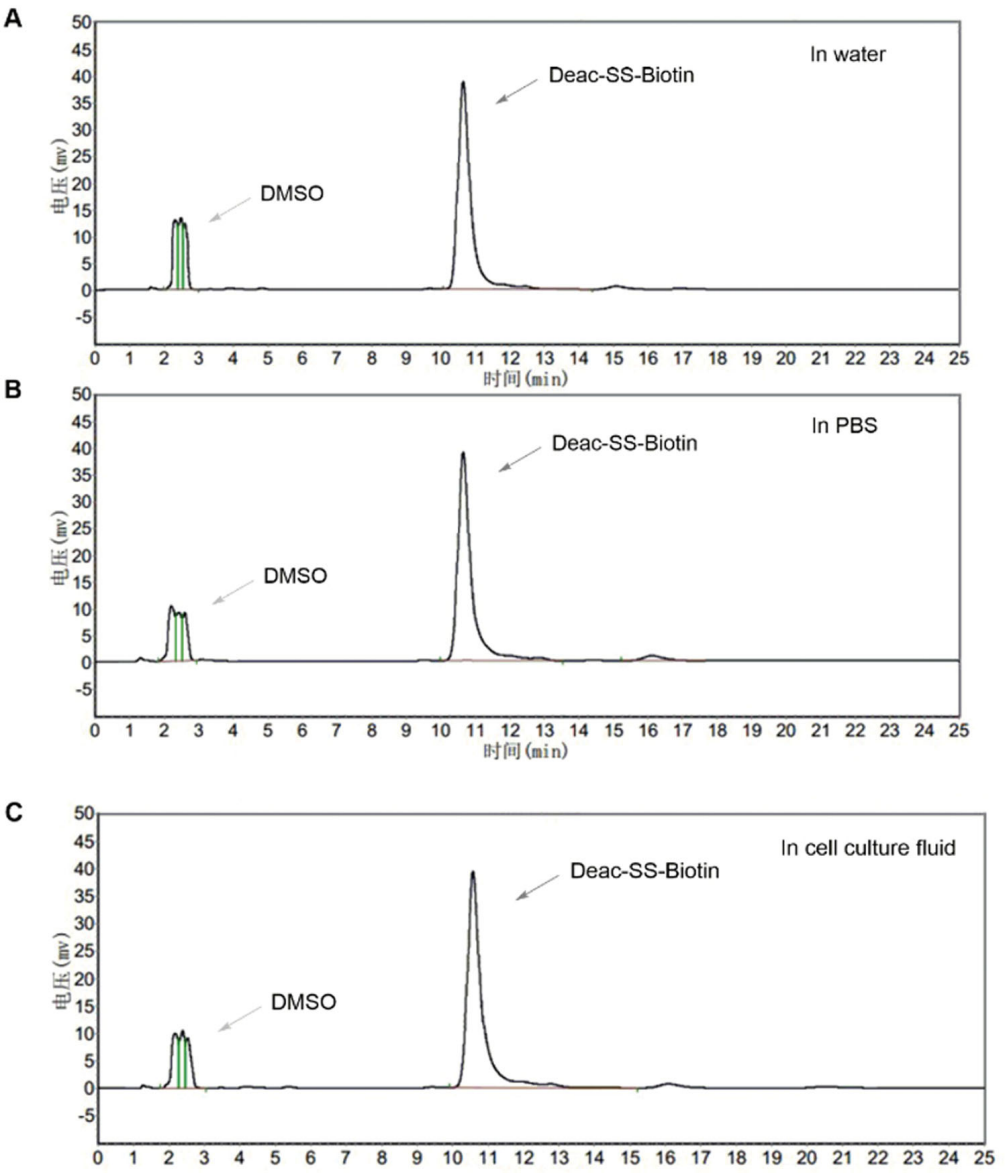
Stabilities of Deac-SS-Biotin (**9**, 5 µM) in water, and PBS cell culture fluid were investigated by HPLC analysis. (A) HPLC analysis of Deac-SS-Biotin (**9**) in water after incubation for and 72 h; (B) HPLC analysis of Deac-SS-Biotin (**9**) in PBS after incubation for and 72 h; (C) HPLC analysis of Deac-SS-Biotin (**9**) in cell culture fluid after incubation for and 72 h.

To confirm that Deac-SS-Biotin (**9**) did not decompose under physiological conditions, the drug stabilities in water, PBS, and, cell culture fluid were investigated by HPLC analysis. The results show that the peak area and peak height of Deac-SS-Biotin (**9**) nearly remained unchanged in water, PBS, and cell culture fluid during the 72 h incubation, demonstrating that Deac-SS-Biotin (**9**) existed stably under physiological conditions (Figure 4).

#### 2.1.3. *In vitro* release of Deac from biotin conjugates

**Figure 5.**
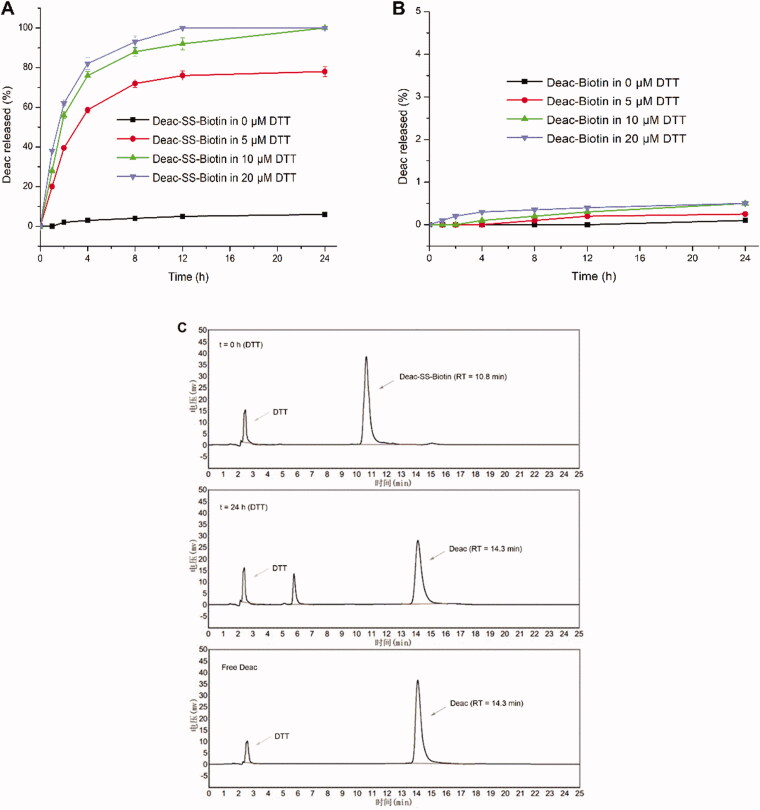
*In vitro* release of Deac from prodrugs. (A) Release profiles of Deac from Deac-SS-Biotin (**9**, 5 μM) with 0 μM, 5 μM, 10 μM and 20 μM DTT in PBS (pH 7.2–7.4) (*n* = 3). (B) Release profiles of Deac from Deac-Biotin (**10**, 5 μM) with 0 μM, 5 μM, 10 μM and 20 μM DTT in PBS (pH 7.2–7.4) (*n* = 3). (C) HPLC spectra of Deac-SS-Biotin (**9**, 5 μM) with 10 μM DTT at 0 and 24 h.

To investigate the mechanism of GSH-triggered drug release, the *in vitro* drug release profiles of Deac-SS-Biotin (**9**) and Deac-Biotin (**10**) were investigated using DTT (Glutathione mimetic). As shown in Figure 5(A), almost none of Deac was released from Deac-SS-Biotin (**9**) within 24 h in blank PBS (pH 7.2–7.4, without DTT), and more than 70% of Deac was released within 8 h in the presence of 5, 10, and 20 μM DTT. By contrast, there was negligible Deac (<1.0%) released from Deac-Biotin (**10**) after 24 h incubation in the presence or absence of 20 μM DTT (Figure 5(B)). The results showed that the drug release from Deac-Biotin (**10**) was extremely slow, and Deac-SS-Biotin (**9**) had a remarkable reduction-responsive drug release. Furthermore, Deac release from the Deac-SS-Biotin (**9**) at 10 μM DTT was also investigated by HPLC analysis (Figure 5(C)). Free Deac-SS-Biotin (**9**) was eluted at 10.8 min in HPLC chromatogram. As 10 μM DTT was added to Deac-SS-Biotin (**9**), it was sheared by DTT. As chemical reaction involved, this scission was subjected to intramolecular cyclisation, followed by ring-forming condensation, resulting in the liberation of Deac. The peak at 14.3 min appeared accompanied by the disappearance of Deac-SS-Biotin (**9**), and well-matched with free Deac. In the light of these data, Deac-SS-Biotin (**9**) existed physiologically and was able to be cleaved by DTT, resulting in the liberation of Deac.

### 2.2. Biological evaluation

#### 2.2.1. Antiproliferative activities of biotin conjugates

The *in vitro* antiproliferative activities of Deac-SS-Biotin (**9**) and biotin conjugates (**10** and **11**) were evaluated against three human tumour cell lines (gastric adenocarcinoma SGC-7901 cells, lung adenocarcinoma A549 cells, and cervical carcinoma HeLa cells) and normal cells (L929 cells) using MTT assay with colchicine as reference (Table 1). Specifically, Deac-SS-Biotin (**9**) exerted potent antiproliferative activity on SGC-7901, A549, and HeLa cell lines with IC_50_ of 0.124 ± 0.011, 0.085 ± 0.008, and 0.108 ± 0.010 μM, respectively, which was close to positive control colchicine and parent drug Deac (**13**).

**Table 1.** Antiproliferative activity of all compounds.

Compounds	IC_50_^a^ (μM) ± SD				
	SGC-7901	A549	Hela	L929	Selectivity (L929/A549)
**9**	**0.124 ± 0.011**	**0.085 ± 0.008**	**0.108 ± 0.010**	**4.22 ± 0.102**	**49**
**10**	4.90 ± 0.18	4.85 ± 0.13	6.35 ± 0.11	15.8 ± 0.31	3
**11a**	7.52 ± 0.12	6.52 ± 0.19	3.50 ± 0.15	>20	–
**11 b**	2.29 ± 0.09	2.23 ± 0.12	6.94 ± 0.16	13.4 ± 0.18	6
**11c**	1.95 ± 0.071	1.31 ± 0.092	2.17 ± 0.10	10.7 ± 0.20	8
**13**	0.069 ± 0.005	0.063 ± 0.004	0.078 ± 0.005	0.131 ± 0.010	2
**4** ^b^	0.066 ± 0.004	0.076 ± 0.006	0.054 ± 0.007	0.164 ± 0.013	2

^a^IC_50_: the half maximal inhibitory concentration.

^b^Used as positive control.

Deac-Biotin (**10**) and Deac-CC-Biotins (**11**) exerted poor activity against the above three human tumour cell lines compared with Deac-SS-Biotin. This might be because of the release of Deac (**13**) from Deac-SS-Biotin (**9**) through the cleavage of the disulphide linkage, while Deac-Biotin (**10**) and Deac-CC-Biotins (**11**) were completely unable to liberate Deac (**13**) in tumour cell lines. However, it was worth noting that the colchicine and Deac (**13**) still exerted potent cytotoxicity towards normal cells L929, while Deac-SS-Biotin (**9**) displayed much lower cytotoxicity comparing with colchicine and Deac (**13**). Meanwhile, the selectivity index of Deac-SS-Biotin (**9**) on SGC-7901, A549, and HeLa cell lines were 34, 49, and 42, respectively, which were much higher than that of colchicine and Deac (**13**). Moreover, Deac-Biotin (**10**) and Deac-CC-Biotins (**11**) also displayed apparently lower cytotoxicity comparing with the parent drug. As a result, Deac-SS-Biotin (**9**) was proved to be a fairly high selectivity conjugate, based on the potent activity on tumour cells, and the good selectivity between tumour and normal cell lines.

**Figure 6.**
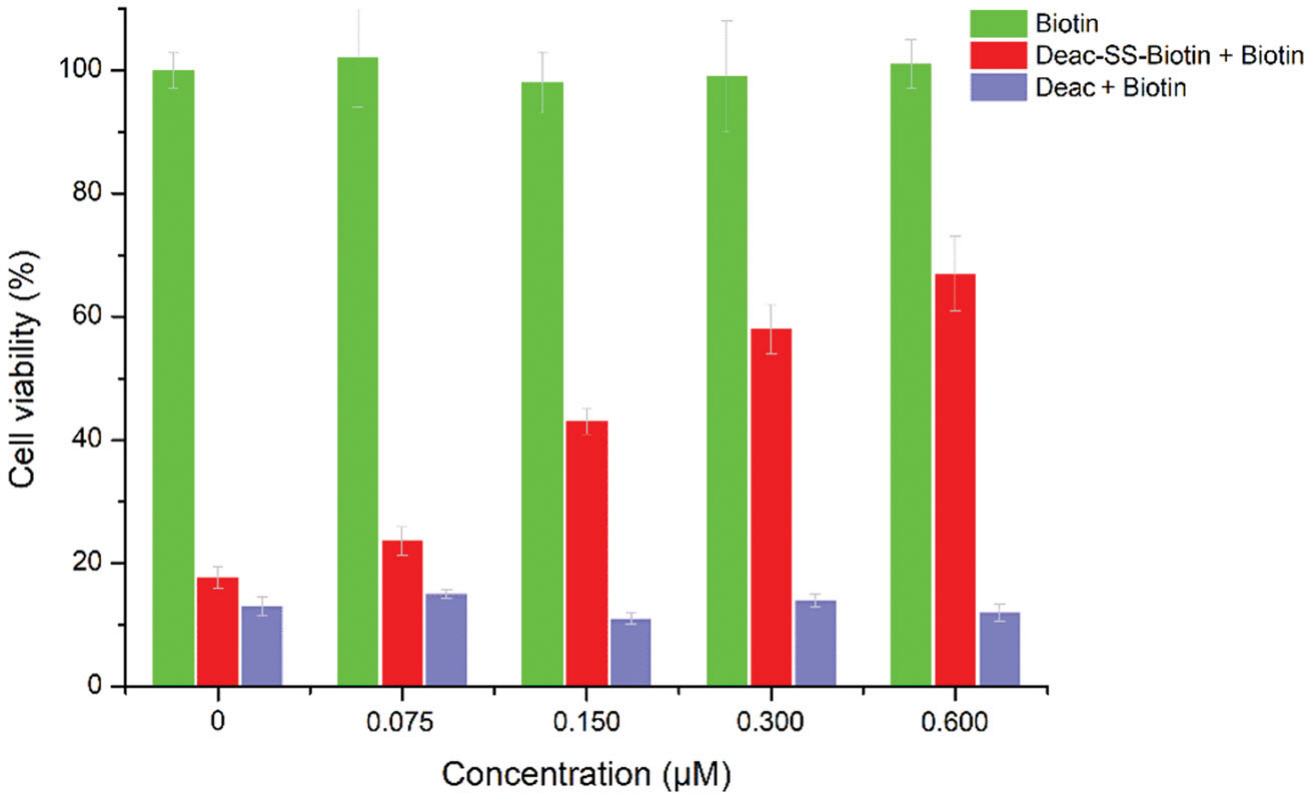
*In vitro* cytotoxicity of biotin, Deac (**13**), and Deac-SS-Biotin (**9**) in A549 cells. Cytotoxicity of biotin, Deac + biotin, and Deac-SS-Biotin + biotin in A549 cells (*n* = 3).

To explore the contribution of biotin receptor to the transport of biotin conjugates, Deac-SS-Biotin (**9**) and biotin were evaluated for the antiproliferative activity against A549 cells. As shown in Figure 6, the cell viability of Deac-SS-Biotin (**9**, 2-fold IC_50_) increased (from 23.6% to 68.9%) with the increase of biotin concentration (from 0.075 to 0.60 μM). In the absence of biotin, the cell viability of Deac-SS-Biotin (**9**) was 17.7%, which was approximate one quarter-fold of 0.6 μM of biotin. The high efficacy of Deac-SS-Biotin (**9**) was quantitatively inhibited by excess biotin, which indicated that biotin effectively prevented Deac-SS-Biotin (**9**) from binding to biotin receptor, thereby blocking the cellular uptake of Deac-SS-Biotin (**9**). By contrast, the cell viability of Deac (**13**, 2-fold IC_50_) remained almost unchanged with the increased concentration of biotin, which suggested that the cell uptake of the Deac (**13**) was not dominated by biotin receptor. In addition, biotin alone caused no cytotoxicity even at various concentrations, suggesting biotin did not affect the cell function. The results showed that the Deac-SS-Biotin’s cellular uptake occurred through biotin-mediated internalisation into the cell.

#### 2.2.2. Tubulin polymerisation inhibition of Deac-SS-Biotin

**Figure 7.**
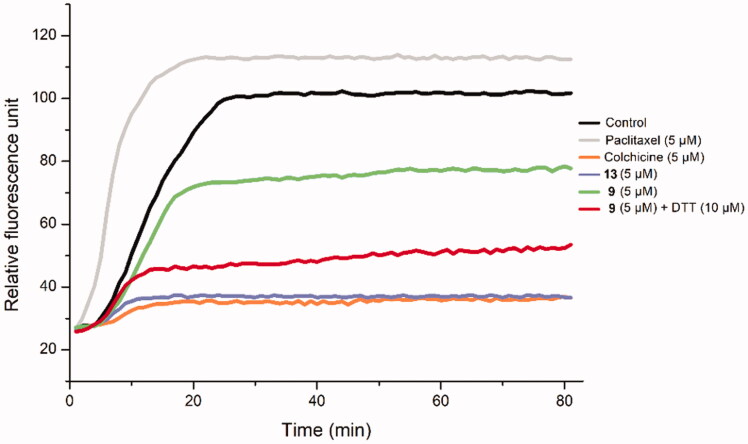
The effect of Deac-SS-Biotin (**9**) on tubulin polymerisation. The tubulin had been pre-incubated for 1 min with Deac (**13**) at 5 µM, Deac-SS-Biotin (**9**) at 5 µM, Deac-SS-Biotin (**9**) at 5 µM and DTT at 10 µM, Deac (**13**) at 5 µM, Colchicine at 5 µM, Paclitaxel at 5 µM or vehicle DMSO at room temperature before GTP was added to start the tubulin polymerisation reactions. The reaction was monitored at 37 °C.

To examine the biological mechanism of biotin conjugates, Deac-SS-Biotin (**9**) was evaluated for its effects on tubulin polymerisation (Figure 7). The results indicated that Deac-SS-Biotin (**9**) had weak inhibition activity on tubulin polymerisation. Following the coincubation of Deac-SS-Biotin (**9**) with DTT, like the positive control colchicine and the parent drug Deac, the product exhibited strong anti-tubulin polymerisation activity. These results provided sufficient evidence that DTT facilitated the transformation of Deac-SS-Biotin (**9**) into Deac (**13**), and this effectively inhibited microtubule assembly and displayed greater antitumor activity.

## 3. Conclusion

In summary, we developed a new tumour-specific conjugate Deac-SS-Biotin that was constituted by cytotoxic agent Deac, biotin, and cleavable disulphide linker. Four nonsensitive biotin-conjugates were synthesised as the counterparts of Deac-SS-Biotin. The Deac-SS-Biotin remained stable in physiological circulation but released active drug Deac following disulphide reduction with DTT. *In vitro* antiproliferative activity showed that Deac-SS-Biotin displayed high toxicity to three tumour cells and lower injuries to nontarget cells. Biotin might enhance the transport capability and selectivity of Deac-SS-Biotin to tumour cells *via* biotin receptor-mediated endocytosis. The tubulin polymerisation assay suggested that Deac-SS-Biotin (with DTT) effectively inhibited tubulin polymerisation. Our findings give new insight into the biotin receptor-targeted delivery of chemotherapeutics and provide an opportunity for the development of novel antitumor drugs.

## 4. Experimental

### 4.1. Chemistry

#### 4.1.1. Materials and methods

Unless otherwise noted, all reagents and solvents were obtained from commercially available sources and were used without purification. Reactions were monitored by TLC with silica gel plates under UV light (254 and 365 nm). The melting point was measured (uncorrected) on a hot-stage microscope (Beijing Taike, X-4). ^1^H NMR and ^13 ^C NMR spectra were tested in CDCl_3_ with TMS as the internal reference on a Bruker AVANCE 400 or 600 (^1^H at 400 or 600 MHz, ^13 ^C at 150 MHz). Mass spectra (MS) were obtained from Agilent Co. Ltd. on an Agilent 1100-sl mass spectrometer with an electrospray ionisation source. HPLC (HPLC-LC-20AT, Shimadzu) using SPD-20A UV as the detector, C18 (4.6 × 250 mm, 0.45 mm) as HPLC analysis column. Method: gradient: 45% methanol in water as mobile elution, flow rate: 1.0 ml min^−1^.

#### 4.1.2. Synthesis of 2-((2-hydroxyethyl)disulfanyl)ethyl (4-nitrophenyl) carbonate (15)

To a solution of 2,2′-disulfanediyldiethanol (1.0 g, 6.5 mmol) and triethylamine (1.0 ml, 6.5 mmol) in THF (10 ml) was added 4-nitrophenyl carbonochloridate (1.2 g, 5.8 mmol). The reaction mixture was stirred for 6 h at room temperature. The resulted white precipitant was removed by filtration and the filtrate was concentrated with rotary evaporation. Then, the concentrated solution was charged with 20 ml DCM, washed sequentially with saturated NaHCO_3_ solution (3 × 25 ml), and brine (3 × 20). The organic layer was collected, dried with anhydrous Na_2_SO_4_, and concentrated to get the crude product. Finally, the crude product was purified by column chromatography with hexane/ethyl acetate (3:1) to get the desired material (0.87 g, yield 43.0%).

#### 4.1.3. Synthesis of 2-((2-hydroxyethyl)disulfanyl)ethyl (S)-(2,3,4,10-tetramethoxy-9-oxo-5,6,7,9-tetrahydrobenzo[a]heptalen-7-yl)carbamate (16)

Deac (1.5 g, 4.1 mmol) in 5 ml THF was added dropwise into the THF solution (10 ml) of **15** (0.87 g, 2.7 mmol) which was left to stir at room temperature for 4 h. Then the solvent was removed by rotary evaporation. The obtained residue was dissolved in 15 ml DCM, which was washed with saturated NaHCO_3_ solution (3 × 25 ml), brine (3 × 20 ml), and dried with anhydrous Na_2_SO_4_ overnight. After removing the solvent, the resulted crude product was purified by column chromatography with hexane/ethyl acetate to achieve **16**. Yellow solid; yield: 63.0%; m.p. 101.3–102.3 °C; ^1^H NMR (300 MHz, CDCl_3_): 7.59 (1H, s), 7.34 (1H, d, *J* = 10.87 Hz), 6.88 (1H, d, *J* = 10.19 Hz), 6.55 (1H, s), 6.16 (1H, m), 4.44 (1H, m), 4.27 (1H, m), 4.17 (1H, m), 4.01 (3H, s), 3.94 (3H, s), 3.91 (3H, s), 3.87 (2H, m), 3.63 (3H, s), 2.88 (2H, m), 2.86 (2H, m), 2.54 (1H, m), 2.39 (1H, m), 2.31 (1H, m), 1.86 (1H, m); MS (ESI) m/z 560.4 [M + Na]^+^.

#### 4.1.4. Synthesis of deacetylcolchicine derivatives (17)

Deac (1 mmol) was dissolved under N_2_ in dry DMSO (2 ml) and treated with corresponding anhydrides (1.1 mol) and *N*-methylmorpholine (4 mmol). After being stirred at room temperature for 45 min, the reaction mixture was worked up (extraction with EtOAc). Column chromatography on silica gel (CH_2_Cl_2_–MeOH, 9:1) afforded compound **17**.

##### 4.1.4.1. Synthesis of (S)-4-oxo-4-((2,3,4,10-tetramethoxy-9-oxo-5,6,7,9-tetrahydrobenzo[a]heptalen-7-yl)amino)butanoic acid (17a)

White solid; yield: 82.5%; m.p. 113.1–115.2 °C; ^1^H NMR (300 MHz, CDCl_3_): 10.07 (1H, s), 7.87 (1H, s), 7.68 (1H, d, *J* = 5.43 Hz), 7.42 (1H, d, *J* = 10.87 Hz), 6.95 (1H, d, *J* = 10.95 Hz), 6.55 (1H, s), 4.69 (1H, m), 3.98 (3H, s), 3.94 (3H, s), 3.90 (3H, s), 3.64 (3H, s), 2.63 (4H, m), 2.51 (1H, m), 2.29 (2H, m), 1.88 (1H, m); MS (ESI) *m/z* 456.3 [M-H]^+^.

##### 4.1.4.2. Synthesis of (S)-5-oxo-5-((2,3,4,10-tetramethoxy-9-oxo-5,6,7,9-tetrahydrobenzo[a]heptalen-7-yl)amino)pentanoic acid (17b)

Yellow solid; yield: 85.0%; m.p. 120.0–122.2 °C; ^1^H NMR (300 MHz, CDCl_3_): 9.04 (1H, s), 7.96 (1H, d, *J* = 5.89 Hz), 7.83 (1H, s), 7.45 (1H, d, *J* = 10.66 Hz), 7.00 (1H, d, *J* = 10.95 Hz), 6.56 (1H, s), 4.66 (1H, m), 4.01 (3H, s), 3.94 (3H, s), 3.91 (3H, s), 3.63 (3H, s), 2.54 (1H, m), 2.42 (2H, m), 2.35 (3H, m), 2.24 (1H, m), 2.03 (1H, m), 1.95 (2H, m); MS (ESI) *m/z* 470.4 [M-H]^+^.

##### 4.1.4.3. Synthesis of (S)-2-((2-oxo-2-((2,3,4,10-tetramethoxy-9-oxo-5,6,7,9-tetrahydrobenzo[a]heptalen-7-yl)amino)ethyl)thio)acetic acid (17c)

Yellow solid; yield: 83.1%; m.p. 114.2–116.2 °C; ^1^H NMR (300 MHz, CDCl_3_): 10.98 (1H, s), 7.82 (1H, d, *J* = 6.34 Hz), 7.71 (1H, s), 7.47 (1H, d, *J* = 10.41 Hz), 7.04 (1H, d, *J* = 11.32 Hz), 6.56 (1H, s), 4.66 (1H, m), 4.03 (3H, s), 3.94 (3H, s), 3.91 (3H, s), 3.61 (3H, s), 3.44 (2H, t, *J* = 14.94 Hz), 3.28 (2H, d, *J* = 2.26 Hz), 2.56 (1H, m), 2.33 (2H, m), 2.00 (1H, m); MS (ESI) *m/z* 489.7 [M-H]^+^.

#### 4.1.5. Synthesis of biotin hydrazine (18)

To a suspension of biotin (150 mg, 0.65 mmol) in MeOH (2 ml) was added SOCl2 (0.15 ml, 2.0 mmol), and the solution was stirred overnight at room temperature to give a clear solution. After evaporation of the solvent and excess SOCl2 under reduced pressure, biotin methyl ester (148 mg) was obtained in 94% yield as a white solid. Biotin methyl ester (148 mg, 0.52 mmol) was dispersed in MeOH (1.5 ml), and hydrazine (0.15 ml, 5 mmol) was added with stirring. After stirring for 16 h, the solution was concentrated under reduced pressure and diluted with water (30 ml). The aqueous layer was washed by chloroform (3 × 25 ml) and concentrated *in vacuo* to give **18** (147 mg, 100% yield) as a white solid. The white solid was used for the next reaction without further purification.

#### 4.1.6. Synthesis of 2-((2-((((S)-2,3,4,10-tetramethoxy-9-oxo-5,6,7,9-tetrahydrobenzo[a]heptalen-7-yl)carbamoyl)oxy)ethyl)disulfanyl)ethyl 5-((3aS,4S,6aR)-2-oxohexahydro-1H-thieno[3,4-d]imidazol-4-yl)pentanoate (9)

Biotin (366 mg, 1.5 mmol), **16** (887 mg, 1.65 mmol) and DMAP (12.2 mg, 0.1 mmol) were dissolved in dry THF (50 ml). DCC (371 mg, 1.8 mmol) was then added and the solution was stirred at room temperature for 20 h. The undissolved material was removed by filtration, and the crude product was purified by silica gel chromatography (CH_2_Cl_2_–MeOH, 7:1) yielding Deac-SS-Biotin (**9**) as a yellow solid. Yellow solid; yield: 72.4%; m.p. 120.1–121.3 °C; ^1^H NMR (300 MHz, CDCl_3_): 7.64 (1H, s), 7.29 (1H, d, *J* = 10.19 Hz), 6.85 (1H, d, *J* = 10.87 Hz), 6.64 (1H, d, *J* = 7.47 Hz), 6.55 (1H, s), 5.84 (2H, s), 4.56 (1H, m), 4.44 (1H, m), 4.37 (2H, m), 4.33 (1H, m), 4.24 (1H, m), 4.18 (1H, m), 4.00 (3H, s), 3.94 (3H, s), 3.90 (3H, s), 3.63 (3H, s), 3.18 (1H, m), 2.92 (3H, m), 2.85 (2H, m), 2.77 (1H, d, *J* = 12.23 Hz), 2.52 (1H, m), 2.37 (2H, m), 2.28 (1H, m), 1.90 (1H, m), 1.76 (1H, m), 1.69 (4H, m), 1.47 (2H, m); ^13 ^C NMR (150 MHz, CDCl_3_): 179.6, 173.6, 164.0, 163.8, 155.3, 153.5, 151.6, 151.1, 141.5, 136.4, 135.2, 134.4, 131.4, 125.5, 112.5, 107.4, 62.4, 62.0, 61.8, 61.4, 61.3, 60.3, 56.4, 56.1, 55.3, 53.8, 40.6, 37.7, 37.1, 37.0, 33.9, 30.0, 28.2, 28.1, 24.7; MS (ESI) *m/z* 764.4 [M + H]^+^.

#### 4.1.7. Synthesis of 5-((3aS,4S,6aR)-2-oxohexahydro-1H-thieno[3,4-d]imidazol-4-yl)-N-((S)-2,3,4,10-tetramethoxy-9-oxo-5,6,7,9-tetrahydrobenzo[a]heptalen-7-yl)pentanamide (10)

Deac (200 mg, 0.56 mmol), biotin (151 mg, 0.62 mmol), HATU (213 mg, 0.56 mmol), and triethylamine (113 mg, 1.12 mmol) were stirred in 5 ml DCM for 8 h. The mixture was washed with 10% citric acid followed by brine and then concentrated *in vacuo*. The crude product was purified by column chromatography. Yellow solid; yield: 91.0%; m.p. 163.4–164.5 °C; ^1^H NMR (300 MHz, CDCl_3_): 7.86 (1H, d, *J* = 7.70 Hz), 7.64 (1H, s), 7.56 (1H, s), 7.26 (1H, d, *J* = 9.96 Hz), 7.13 (1H, s), 6.82 (1H, d, *J* = 11.32 Hz), 6.46 (1H, s), 4.64 (1H, m), 4.56 (1H, m), 4.32 (1H, m), 3.88 (3H, s), 3.86 (3H, s), 3.83 (3H, s), 3.58 (3H, s), 3.08 (1H, m), 2.85 (1H, m), 2.69 (1H, d, *J* = 13.13 Hz), 2.42 (1H, m), 2.23 (2H, m), 2.09 (2H, m), 1.91 (1H, m), 1.70 (1H, m), 1.58 (3H, m), 1.36 (2H, m); ^13 ^C NMR (150 MHz, CDCl_3_): 179.7, 173.2, 165.1, 163.9, 153.5, 152.7, 151.1, 141.6, 137.2, 135.4, 134.4, 131.2, 125.6, 113.1, 107.5, 61.6, 61.4, 61.3, 60.7, 56.3, 56.1, 56.0, 51.7, 40.7, 37.0, 35.9, 30.1, 28.4, 28.2, 26.4; MS (ESI) m/z 584.3 [M + H]^+^.

#### 4.1.8. Synthesis of Deac-CC-Biotin (11)

Triethylamine (1.0 mmol) was added to a solution of biotin hydrazine (1.0 mmol) and **17** (1.0 mmol) in dry DMF (5 ml) and the mixture stirred for 10 min at ambient temperature; then to the mixture were added EDCI (1.1 mmol), HOBt (1.1 mmol), and DMAP (0.05 mmol), and the mixture was stirred for 10 h at room temperature. The reaction was quenched with water and the mixture was diluted with EtOAc and extracted with EtOAc. The organic layers were washed with water and dried over Na_2_SO_4_, and the solvent was evaporated *in vacuo*. The residue was purified by recrystallization with EtOAc to afford **11**.

##### 4.1.8.1. Synthesis of 4-oxo-4–(2-(5-((3aS,4S,6aR)-2-oxohexahydro-1H-thieno[3,4-d]imidazol-4-yl)pentanoyl)hydrazinyl)-N-((S)-2,3,4,10-tetramethoxy-9-oxo-5,6,7,9-tetrahydrobenzo[a]heptalen-7-yl)butanamide (11a)

Yellow solid; yield: 61.1%; m.p. 142.2–143.7 °C; ^1^H NMR (300 MHz, CDCl_3_): 10.33 (1H, s), 9.76 (1H, s), 8.03 (1H, d, *J* = 5.43 Hz), 7.86 (1H, s), 7.36 (1H, d, *J* = 10.87 Hz), 7.03 (1H, s), 6.92 (1H, d, *J* = 10.87 Hz), 6.55 (1H, s), 6.32 (1H, s), 4.58 (1H, m), 4.52 (1H, s), 4.37 (1H, s), 3.99 (3H, s), 3.93 (3H, s), 3.90 (3H, s), 3.64 (3H, s), 3.15 (1H, s), 2.89 (1H, d, *J* = 9.96 Hz), 2.74 (1H, d, *J* = 12.68 Hz), 2.53 (1H, d, *J* = 7.24 Hz), 2.30 (7H, m), 1.99 (2H, s), 1.80 (2H, d, *J* = 6.34 Hz), 1.65 (2H, m), 1.51 (2H, m); ^13 ^C NMR (150 MHz, CDCl_3_): 178.7, 172.8, 171.7, 171.6, 163.8, 162.9, 152.6, 152.2, 150.1, 140.6, 136.6, 134.9, 133.5, 128.7, 124.4, 112.6, 106.5, 60.8, 60.7, 60.3, 59.4, 55.4, 55.2, 54.6, 51.6, 39.5, 35.0, 32.9, 32.2, 30.9, 30.7, 27.0, 26.8, 24.2; MS (ESI) *m/z* 720.2 [M + Na]^+^.

##### 4.1.8.2. Synthesis of 5-oxo-5–(2-(5-((3aS,4S,6aR)-2-oxohexahydro-1H-thieno[3,4-d]imidazol-4-yl)pentanoyl)hydrazinyl)-N-((S)-2,3,4,10-tetramethoxy-9-oxo-5,6,7,9-tetrahydrobenzo[a]heptalen-7-yl)pentanamide (11b)

Yellow solid; yield: 68.2%; m.p. 136.6–137.3 °C; ^1^H NMR (300 MHz, CDCl_3_): 9.80 (1H, s), 9.62 (1H, s), 8.10 (1H, s), 7.47 (1H, s), 7.30 (1H, d, *J* = 11.77 Hz), 6.87 (2H, d, *J* = 10.87 Hz), 6.54 (1H, s), 6.24 (1H, s), 4.55 (1H, s), 4.45 (1H, s), 4.32 (1H, s), 3.96 (3H, s), 3.91 (3H, s), 3.89 (3H, s), 3.62 (3H, s), 3.09 (1H, s), 2.84 (1H, d, *J* = 8.15 Hz), 2.69 (2H, d, *J* = 11.77 Hz), 2.51 (4H, d, *J* = 13.58 Hz), 2.30 (3H, s), 2.19 (2H, s), 1.91 (1H, s), 1.68 (3H, s), 1.57 (3H, d, *J* = 13.58 Hz), 1.43 (1H, s); ^13 ^C NMR (150 MHz, CDCl_3_): 179.7, 173.1, 171.8, 171.7, 164.6, 164.0, 153.5, 152.6, 151.0, 141.5, 137.1, 135.6, 134.5, 130.9, 125.6, 113.1, 107.4, 61.9, 61.6, 61.4, 60.4, 56.4, 56.2, 55.8, 52.3, 40.6, 36.4, 33.4, 31.9, 31.3, 30.0, 29.5, 28.0, 25.4, 22.7; MS (ESI) *m/z* 734.3 [M + Na]^+^.

##### 4.1.8.3. Synthesis of 2-((2-oxo-2–(2-(5-((3aS,4S,6aR)-2-oxohexahydro-1H-thieno[3,4-d]imidazol-4-yl)pentanoyl)hydrazinyl)ethyl)thio)-N-((S)-2,3,4,10-tetramethoxy-9-oxo-5,6,7,9-tetrahydrobenzo[a]heptalen-7-yl)acetamide (11c)

Yellow solid; yield: 70.2%; m.p. 127.3–128.9 °C; ^1^H NMR (300 MHz, CDCl_3_): 10.37 (1H, s), 10.11 (1H, s), 8.44 (1H, d, *J* = 7.24 Hz), 7.67 (1H, s), 7.37 (1H, d, *J* = 9.96 Hz), 6.93 (1H, d, *J* = 10.87 Hz), 6.86 (1H, s), 6.55 (1H, s), 6.29 (1H, s), 4.62 (1H, s), 4.51 (1H, s), 4.36 (1H, s), 3.99 (3H, s), 3.93 (3H, s), 3.90 (3H, s), 3.63 (3H, s), 3.42 (2H, t, *J* = 18.11 Hz), 3.27 (2H, t, *J* = 15.40 Hz), 3.13 (1H, s), 2.88 (1H, d, *J* = 9.96 Hz), 2.73 (1H, d, *J* = 10.87 Hz), 2.53 (1H, d, *J* = 6.34 Hz), 2.36 (4H, s), 2.01 (1H, s), 1.77 (2H, s), 1.63 (2H, s), 1.49 (2H, s); ^13 ^C NMR (150 MHz, CDCl_3_): 179.7, 173.6, 169.0, 168.9, 164.7, 164.0, 153.6, 152.9, 151.1, 141.6, 137.4, 135.9, 134.4, 130.7, 125.4, 113.6, 107.6, 61.8, 61.7, 61.4, 60.4, 56.4, 56.1, 55.7, 52.9, 40.5, 36.2, 35.5, 33.9, 33.2, 30.0, 28.0, 27.8, 25.2; MS (ESI) *m/z* 754.1 [M + Na]^+^.

#### 4.1.9. Physiological stability of Deac-SS-Biotin

To 8 ml of water, PBS, or cell culture fluid, 2 ml of DMSO stock solution of Deac-SS-Biotin was added. The mixture was incubated with gentle shaking for 72 h at room temperature during which the mixture was measured by HPLC spectra.

#### 4.1.10. In vitro release of Deac from biotin conjugates

The release of Deac from Deac-SS-Biotin was studied in PBS (pH 7.2–7.4) containing 20% DMSO with 0, 5, 10, or 20 μM, dithiothreitol (DTT), respectively. Briefly, biotin-conjugates (5 μM) were incubated in 10 ml of release medium at 37 °C. At timed intervals, 0.1 ml of sample was withdrawn, and the content of Deac, Deac-Biotin, and Deac-SS-Biotin was determined by HPLC (HPLC-LC-20AT, Shimadzu using SPD-20A UV as the detector, C18 (4.6 × 250 mm, 0.45 mm) as HPLC analysis column. Method: gradient: 45% methanol in water as mobile elution, flow rate: 1.0 ml min^-^1). The UV detector was kept at 254 nm.

### 4.2. Biological evaluation

#### 4.2.1. Cell culture

The human gastric adenocarcinoma SGC-7901 cells, lung adenocarcinoma A549 cells, cervical carcinoma HeLa cells, and mouse fibroblasts L929 cells were cultured in RPMI-1640 medium containing 10% FBS, 100 U/mL streptomycin, and 100 U/mL penicillin at 37 °C in a humidified atmosphere containing 5% CO_2_. All cell lines were purchased from the American Type Culture Collection (ATCC, Manassas, VA)^29^.

#### 4.2.2. In vitro antiproliferative activity

The *in vitro* anti-proliferative activities of colchicine, Deac, and biotin-conjugates were determined by MTT (Sigma) assay. Briefly, cells were seeded into 96-well plates at a density of 1–3 × 10^4^/well, depending on the growth rate of the cell line. 24 h later, triplicate wells were treated with media and the compounds being tested. After 72 h of incubation at 37 °C in 5% CO_2_, the drug-containing medium was removed and replaced with 100 ml of fresh medium containing 5 mg/mL MTT solution. After 4 h of incubation, the medium with MTT was removed, and 100 ml of dimethyl sulfoxide (DMSO) was added to each well. The plates were gently agitated until the purple formazan crystals were dissolved, and the OD490 values were determined using a microplate reader (MK3, Thermo, Germany). The data were calculated and plotted as the percent viability compared to the control. The 50% inhibitory concentration (IC_50_) was defined as the drug concentration that resulted in an absorbance of 50% of that of the untreated wells in the MTT assay^30^.

#### 4.2.3. *In vitro* tubulin polymerisation assay

The effects of colchicine, Deac, and Deac-SS-Biotin on the polymerisation of tubulin were determined by employing a fluorescence-based tubulin polymerisation assay kit (BK011P, Cytoskeleton, USA) according to the manufacturer’s protocol. The tubulin reaction mix contained 2 mg/mL porcine brain tubulin (>99% pure), 2 mM MgCl_2_, 0.5 mM EGTA, 1 mM GTP, and 15% glycerol. First, 96-well plate was incubated with 5 ml of inhibitors in various concentrations at 37 °C for 1 min. Then 50 ml of the tubulin reaction mix was added. The samples were mixed well, and tubulin assembly was monitored (emission wavelength of 420 nm; excitation wavelength pf 360 nm) at 1 min intervals for 90 min at 37 °C using a plate reader (FAS-Calibur, BD Biosciences, USA). The IC_50_ values were calculated after 20 min using the SPSS software [31].

## Supplementary Material

Supplemental Material
