# Supplementary material for: Design, synthesis, and biological evaluation of biotinylated colchicine derivatives as potential antitumor agents
Source: J Enzyme Inhib Med Chem. 2021 Dec 16;37(1):411–20. doi: 10.1080/14756366.2021.2013832 (PMC8725855; doi:10.1080/14756366.2021.2013832)
Supplement: Supplemental Material [file IENZ_A_2013832_SM4951.pdf]

Design, synthesis, and biological evaluation of biotinylated colchicine derivatives as potential antitumor agents

Chao Wang [a*], Yujing Zhang [b*], Zeyu Wang [c], Yuelin Li [c], Qi Guan [c], Dongming Xing [a*], Weige Zhang [c*]

[a] The Affiliated Hospital of Qingdao University, Qingdao University, Cancer Institute, Qingdao 266071, Shandong, China

[b] The Affiliated Cardiovascular Hospital of Qingdao University, Qingdao University, Qingdao, 266071, Shandong, China

[c] Key Laboratory of Structure-Based Drug Design and Discovery, Ministry of Education, Shenyang Pharmaceutical University, Shenyang 110016, Liaoning, China.

E-mail addresses: wangchao20086925@126.com (C. Wang), 459233223@qq.com (Y. Zhang), zhangweige2000@sina.com (W. Zhang).

Contents:

Mass, ^{1}H-NMR and ^{13}C-NMR spectra of all target compounds:

*2-((2-hydroxyethyl)disulfanyl)ethyl (S)-(2,3,4,10-tetramethoxy-9-oxo-5,6,7,9-tetrahydrobenzo[a]heptalen-7-yl)carbamate (**16**)*

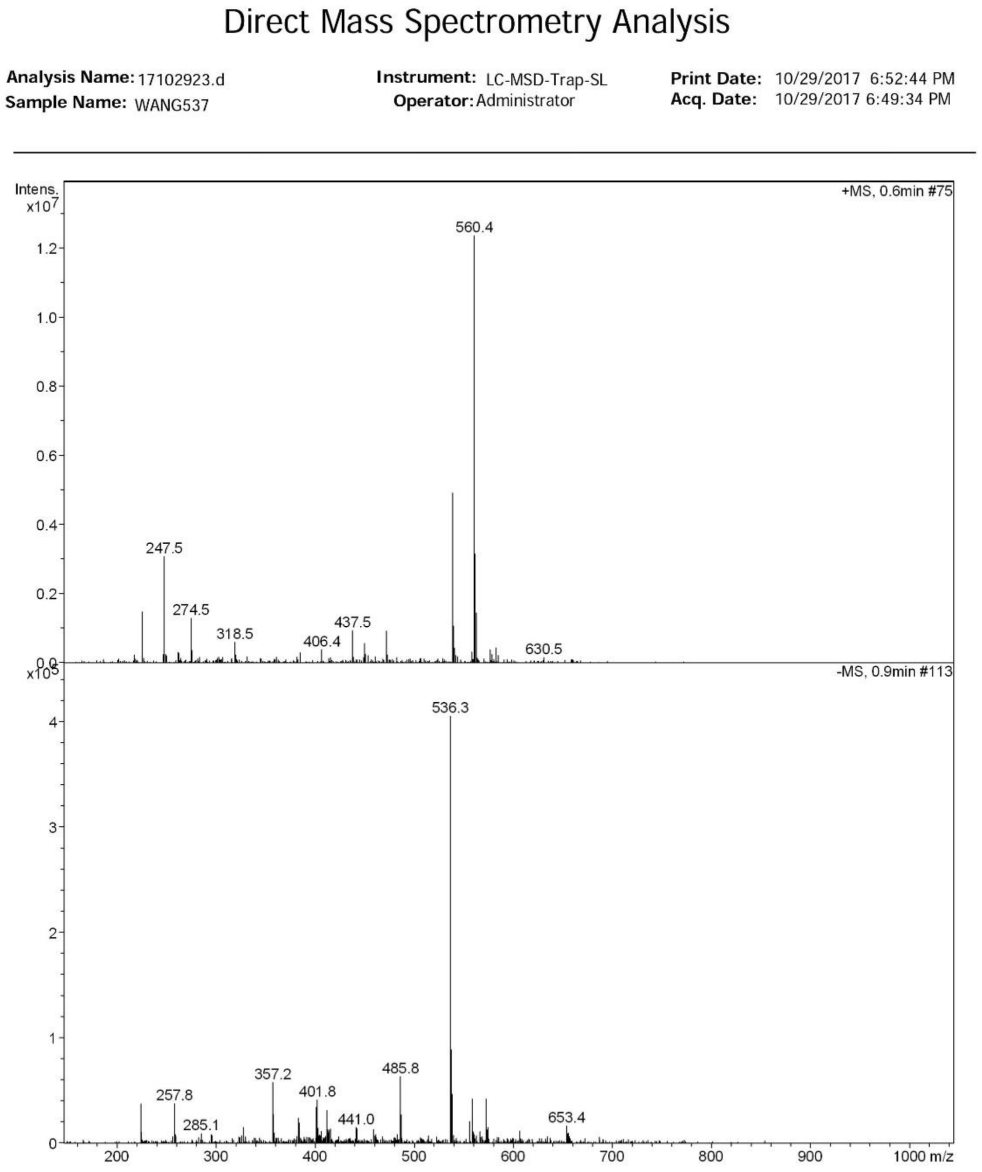

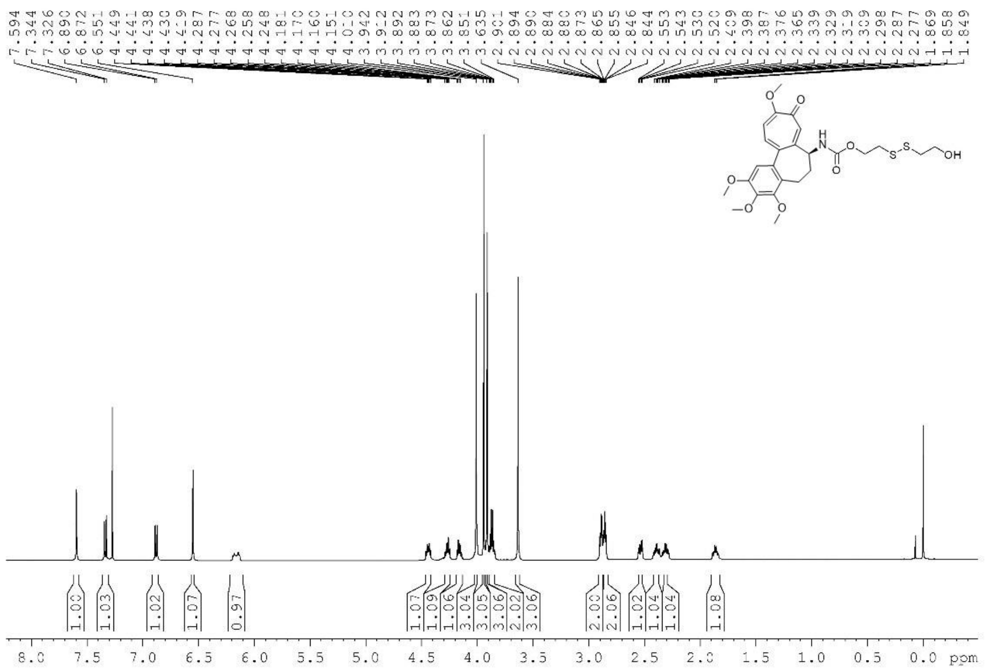

*(S)-4-oxo-4-((2,3,4,10-tetramethoxy-9-oxo-5,6,7,9-tetrahydrobenzo[a]heptalen-7-yl)amino)butanoic acid (**17a**)*

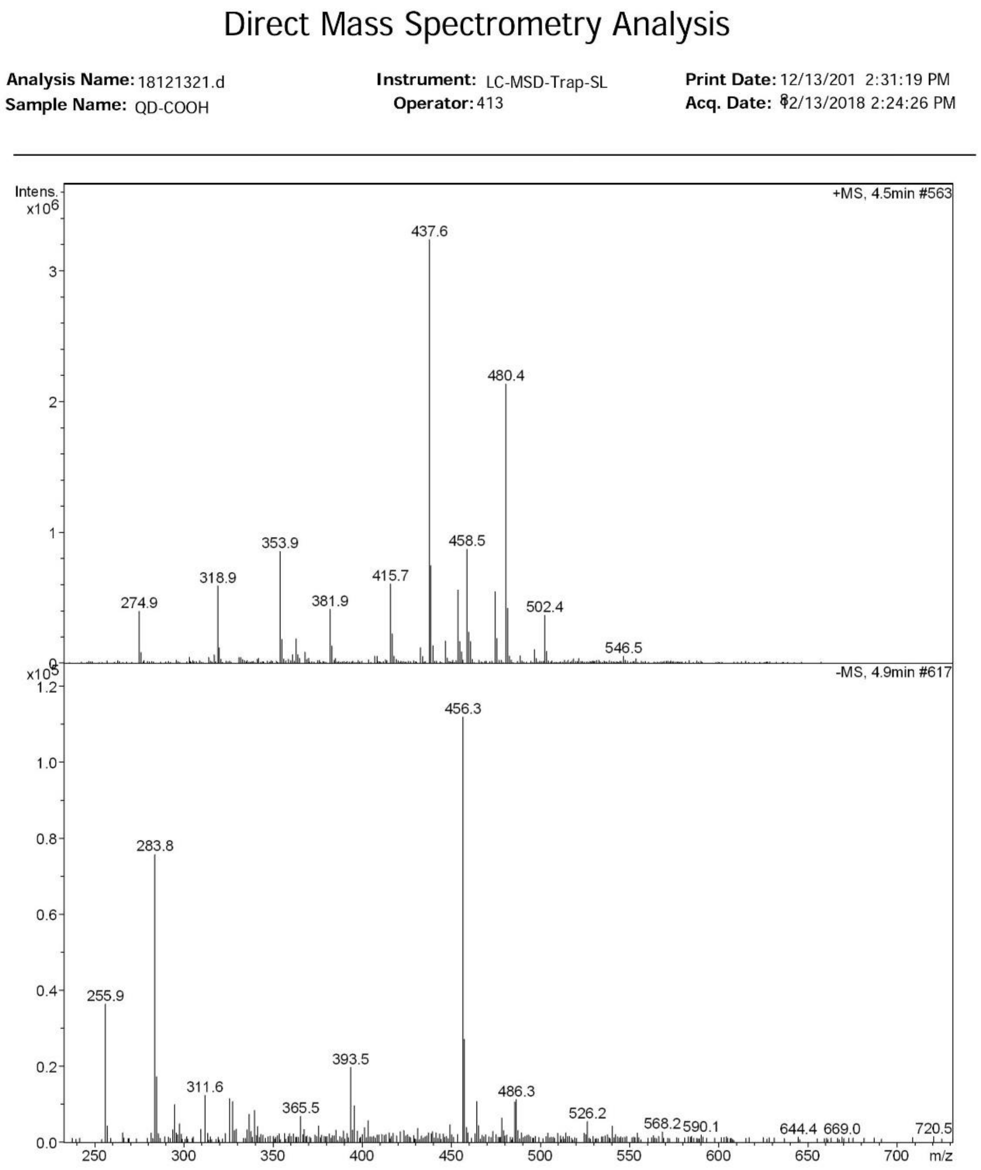

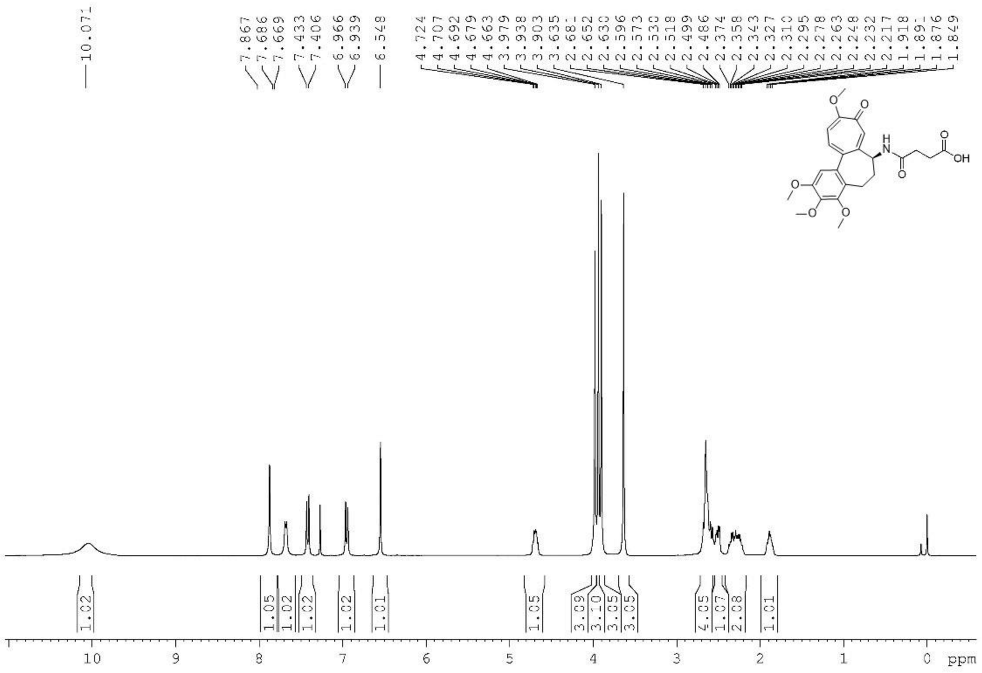

*(S)-5-oxo-5-((2,3,4,10-tetramethoxy-9-oxo-5,6,7,9-tetrahydrobenzo[a]heptalen-7-yl)amino)pentanoic acid (**17b**)*

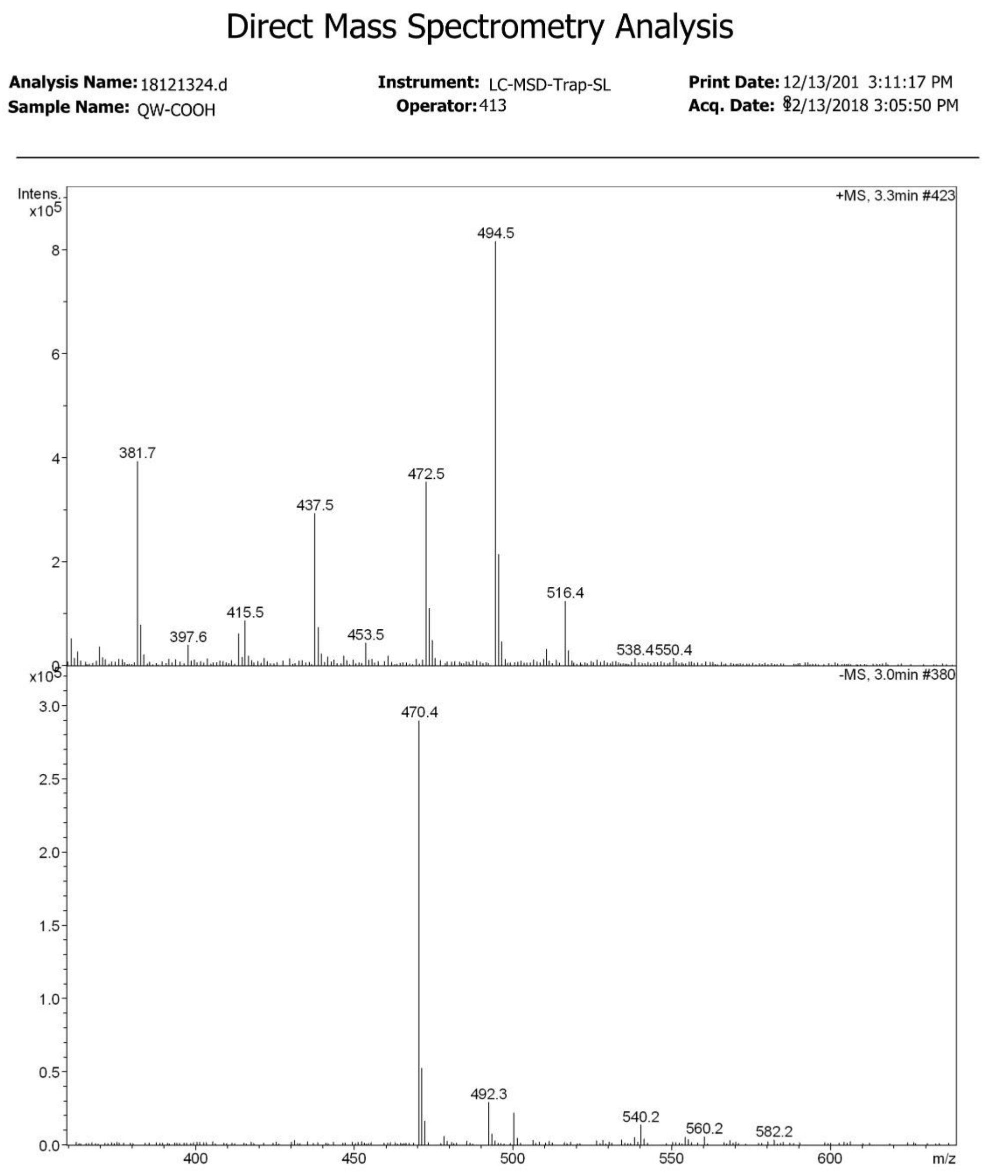

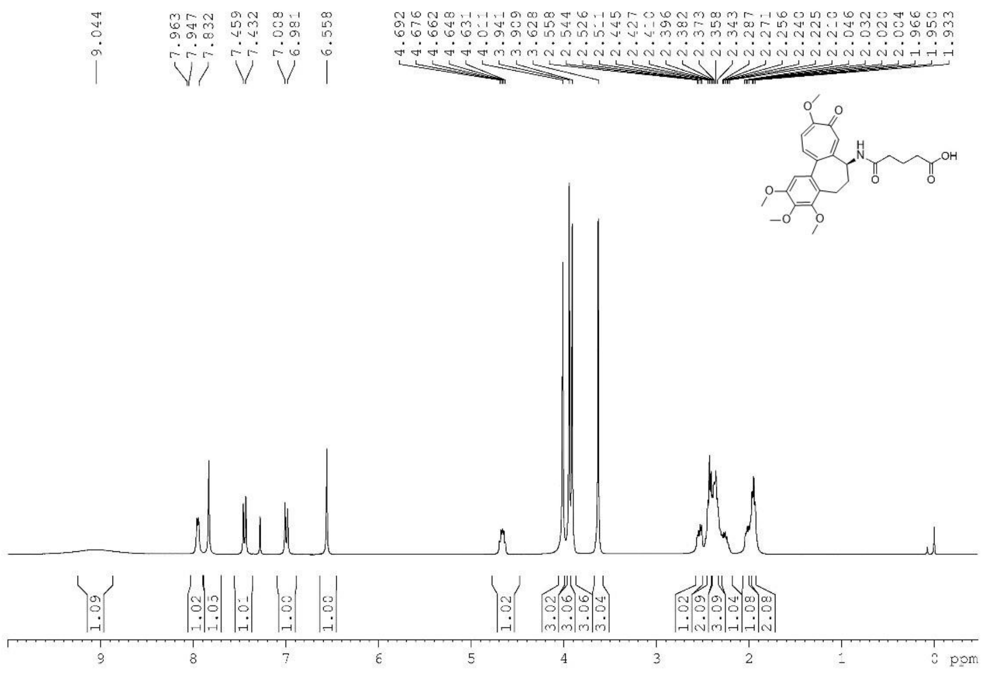

*(S)-2-((2-oxo-2-((2,3,4,10-tetramethoxy-9-oxo-5,6,7,9-tetrahydrobenzo[a]heptalen-7-yl)amino)ethyl)thio)acetic acid (**17c**)*

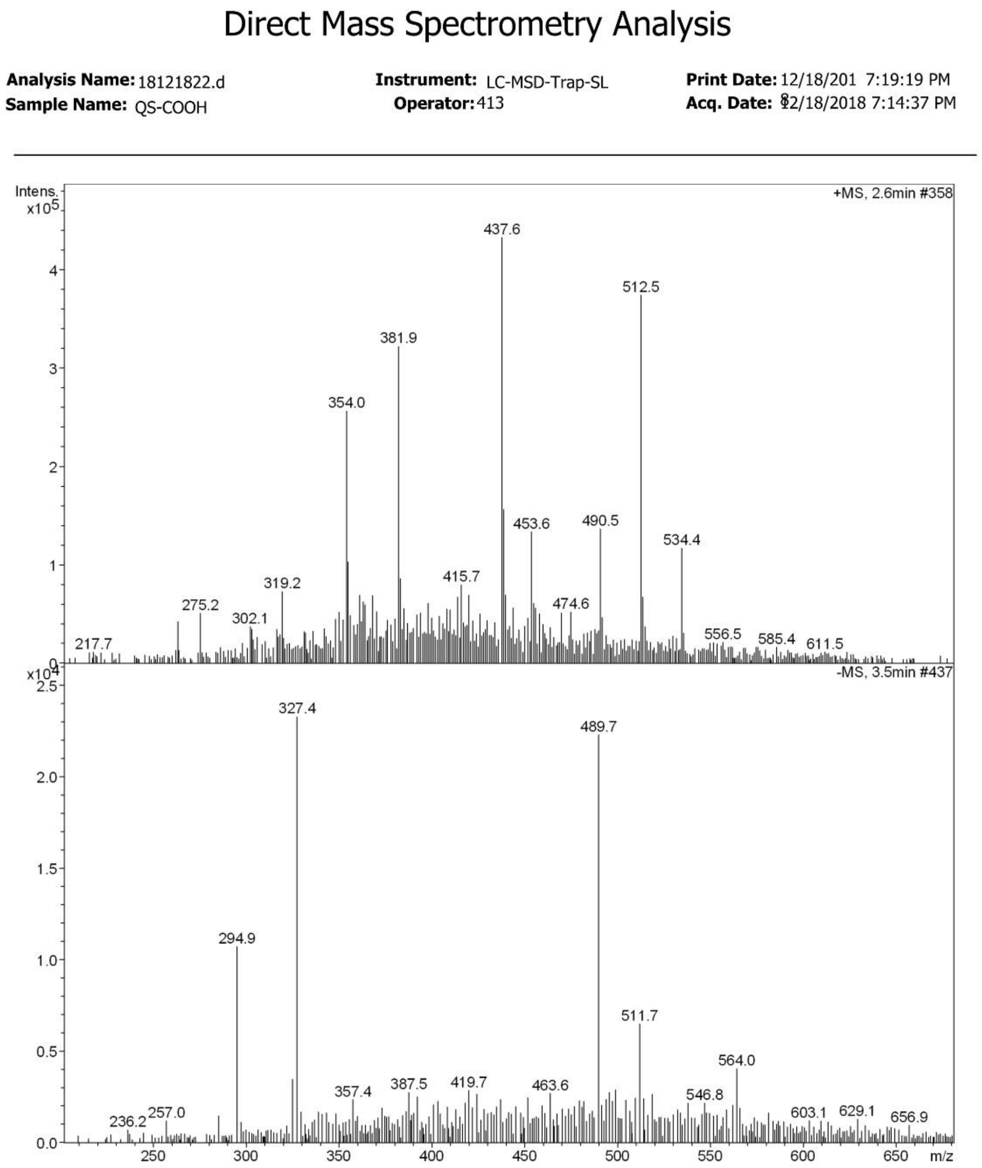

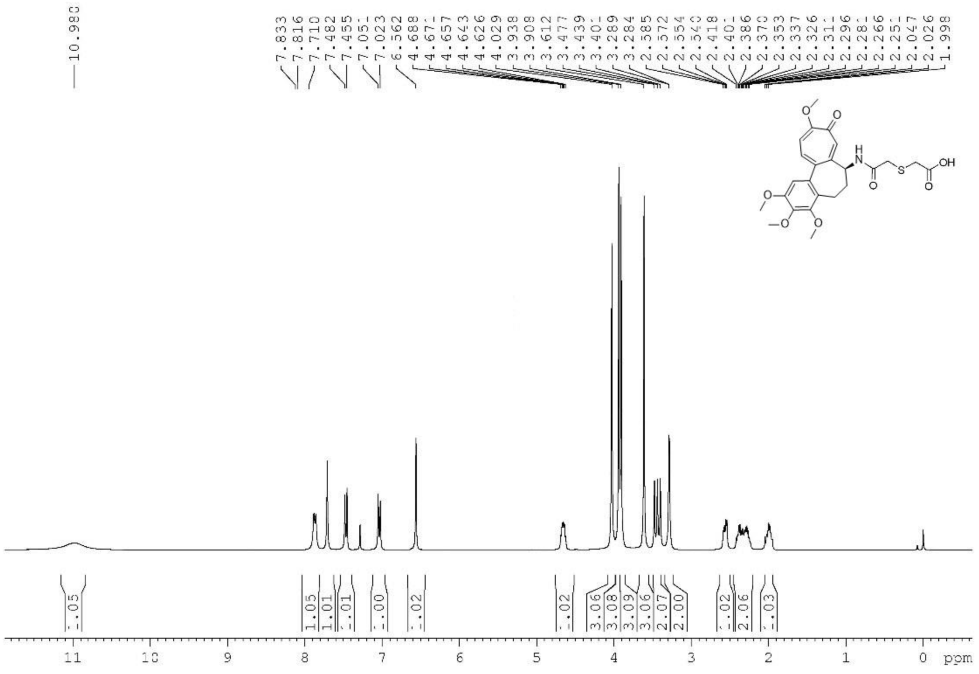

*2-((2-((((S)-2,3,4,10-tetramethoxy-9-oxo-5,6,7,9-tetrahydrobenzo[a]heptalen-7-yl)carbamoyl)oxy)ethyl)disulfanyl)ethyl 5-((3aS,4S,6aR)-2-oxohexahydro-1H-thieno[3,4-d]imidazol-4-yl)pentanoate (**9**)*

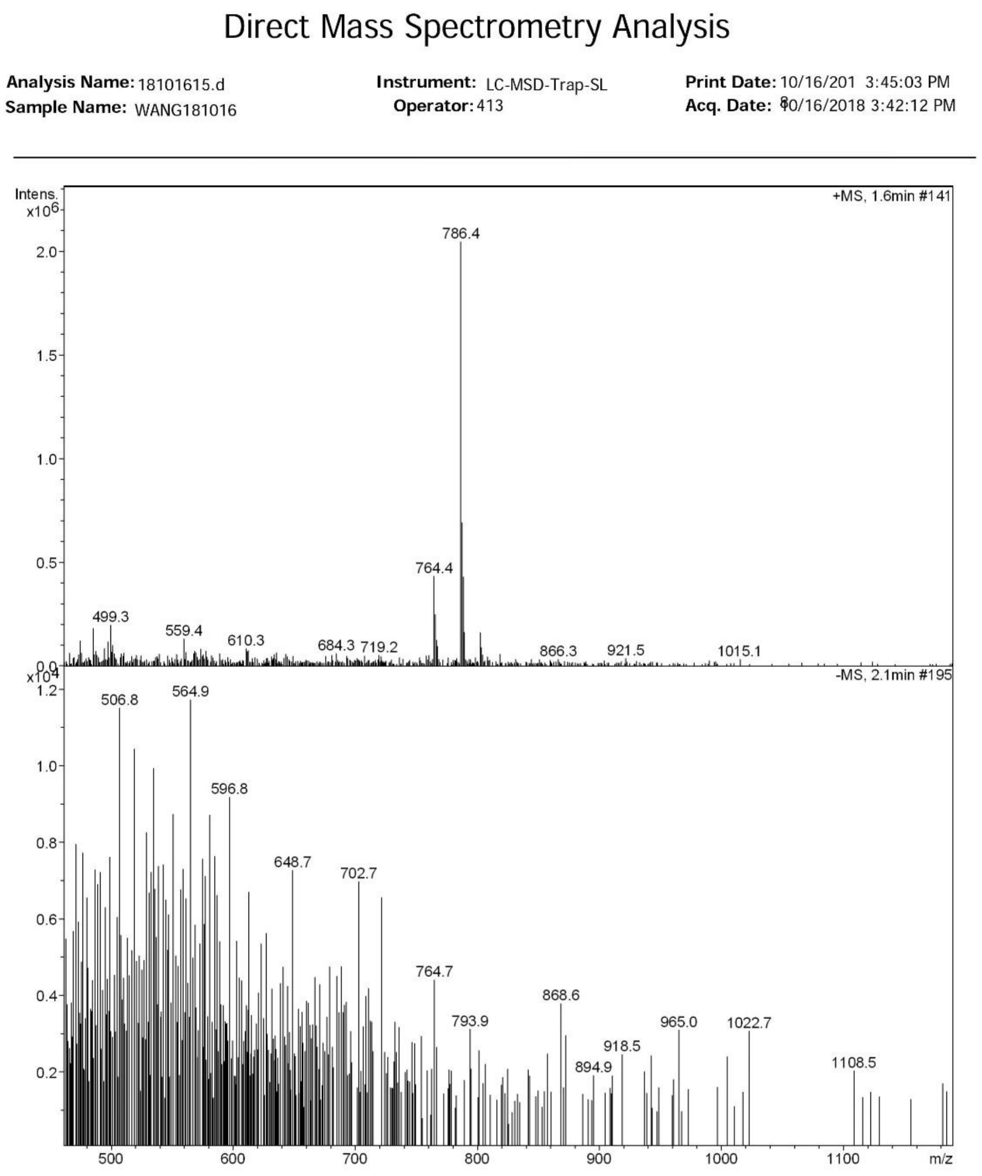

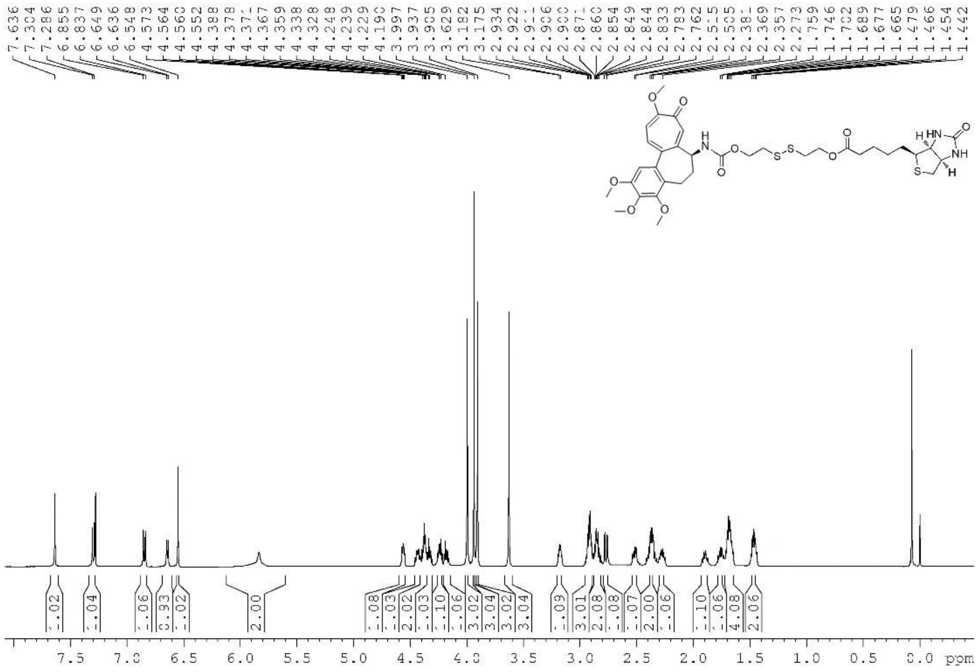

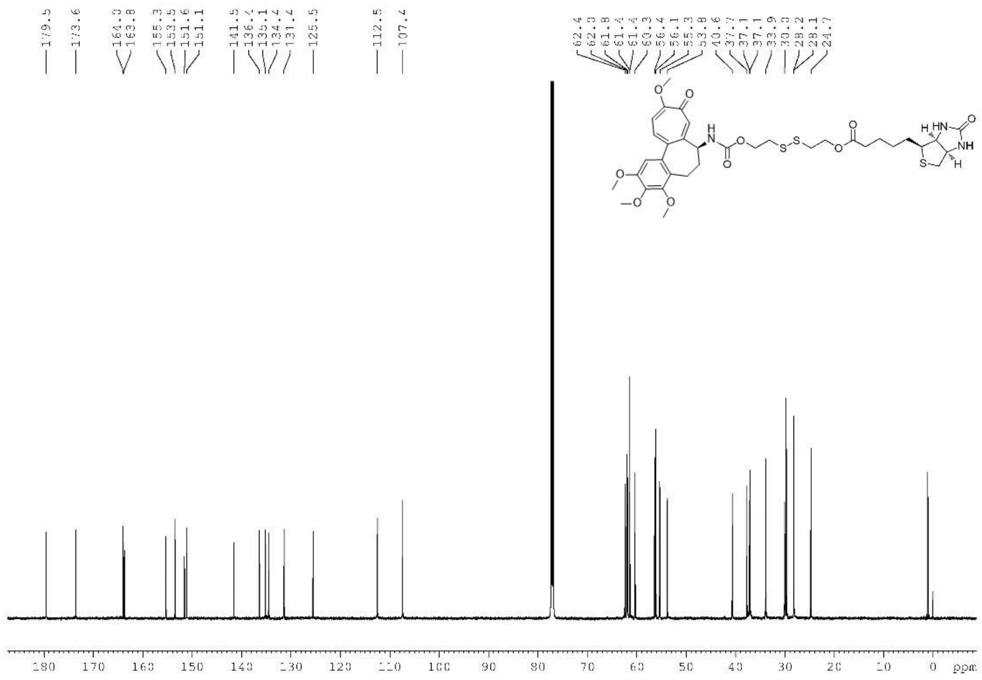

179.5
173.6
164.0
163.8
155.3
153.5
151.6
151.1
141.5
136.4
135.1
134.4
131.4
125.5
112.5
107.4
62.4
62.3
61.8
61.4
61.4
60.3
56.4
56.1
55.3
53.8
40.6
37.7
37.1
37.1
33.9
30.3
28.2
28.1
24.7
180 170 160 150 140 130 120 110 100 90 80 70 60 50 40 30 20 10 0 ppm
HN
NH
S
H
O
N

*5-((3aS,4S,6aR)-2-oxohexahydro-1H-thieno[3,4-d]imidazol-4-yl)-N-((S)-2,3,4,10-tetramethoxy-9-oxo-5,6,7,9-tetrahydrobenzo[a]heptalen-7-yl)pentanamide (**10**)*

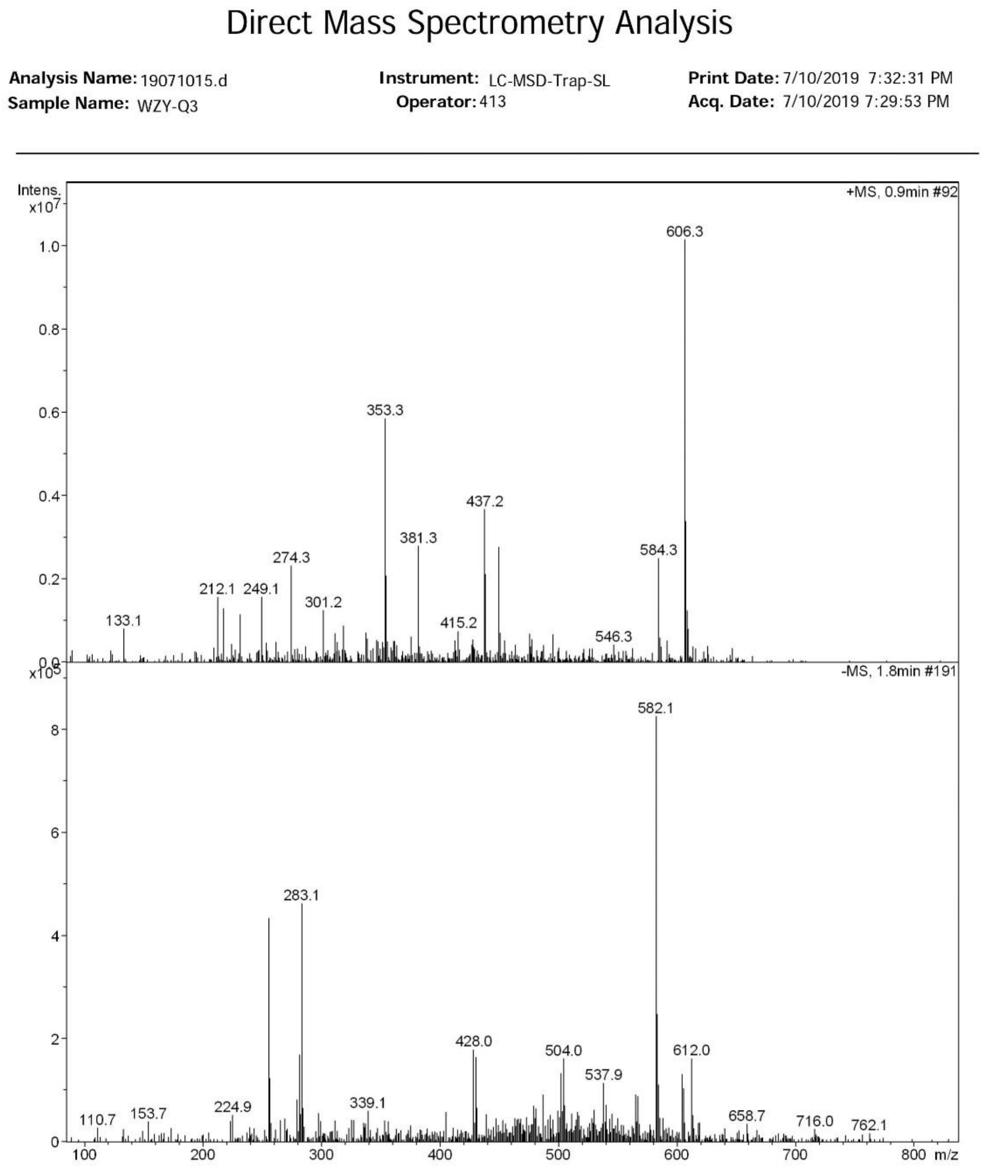

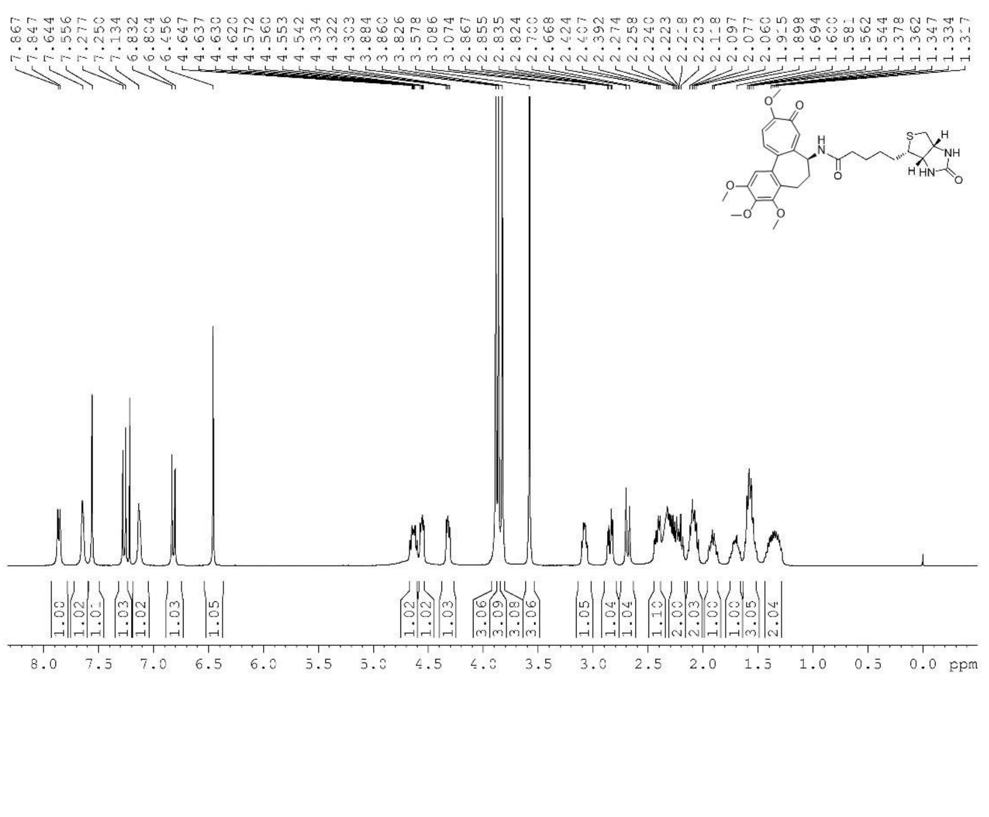

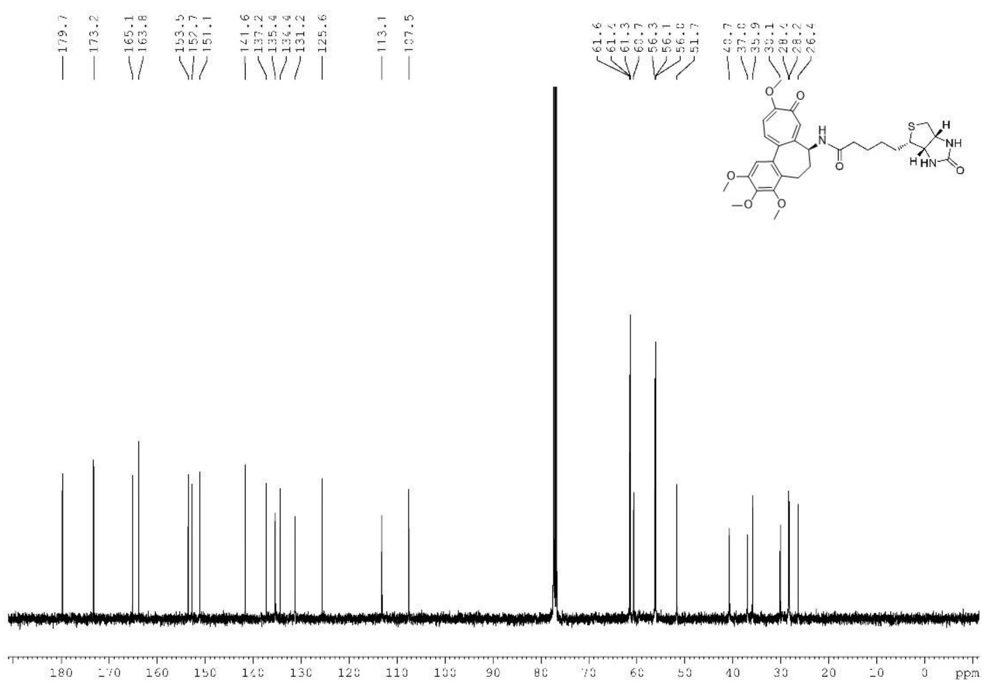

*4-oxo-4-(2-(5-((3aS,4S,6aR)-2-oxohexahydro-1H-thieno[3,4-d]imidazol-4-yl)pentanoyl)hydrazinyl)-N-((S)-2,3,4,10-tetramethoxy-9-oxo-5,6,7,9-tetrahydrobenzo[a]heptalen-7-yl)butanamide (**11a**)*

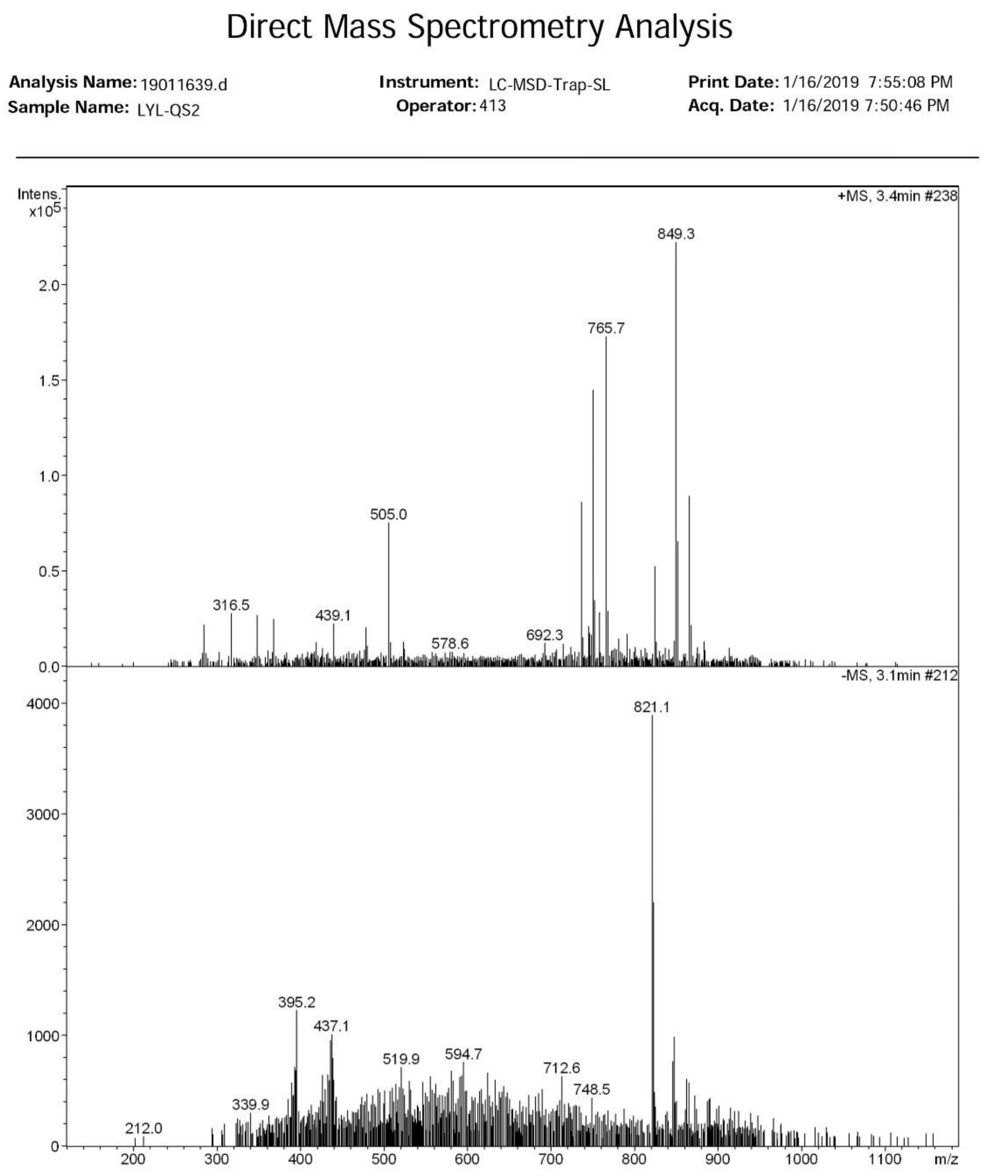

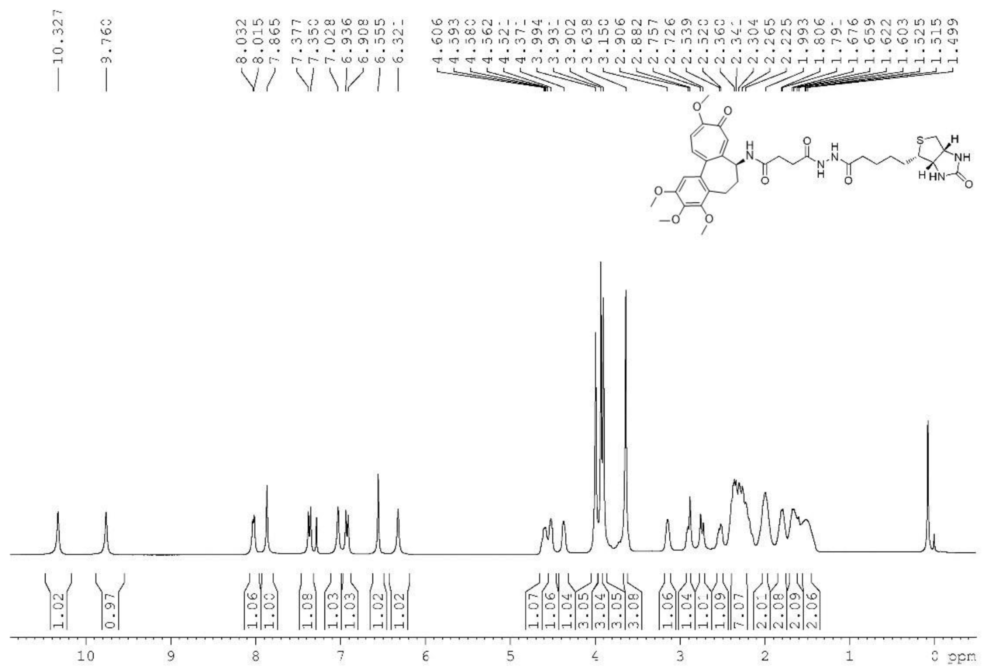

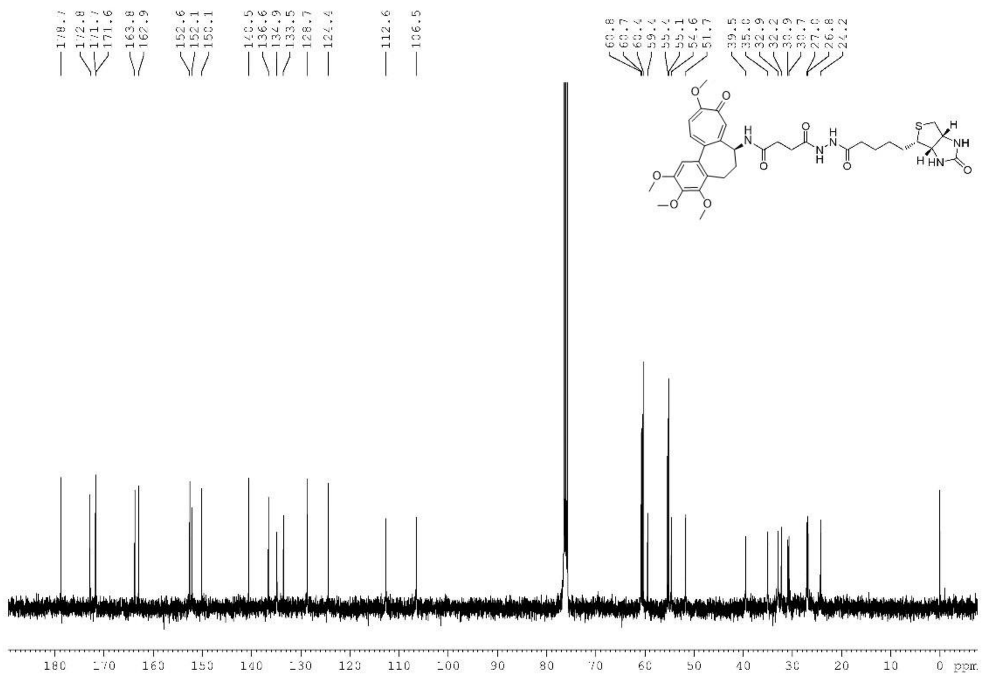

*5-oxo-5-(2-(5-((3aS,4S,6aR)-2-oxohexahydro-1H-thieno[3,4-d]imidazol-4-yl)pentanoyl)hydrazinyl)-N-((S)-2,3,4,10-tetramethoxy-9-oxo-5,6,7,9-tetrahydrobenzo[a]heptalen-7-yl)pentanamide (**11b**)*

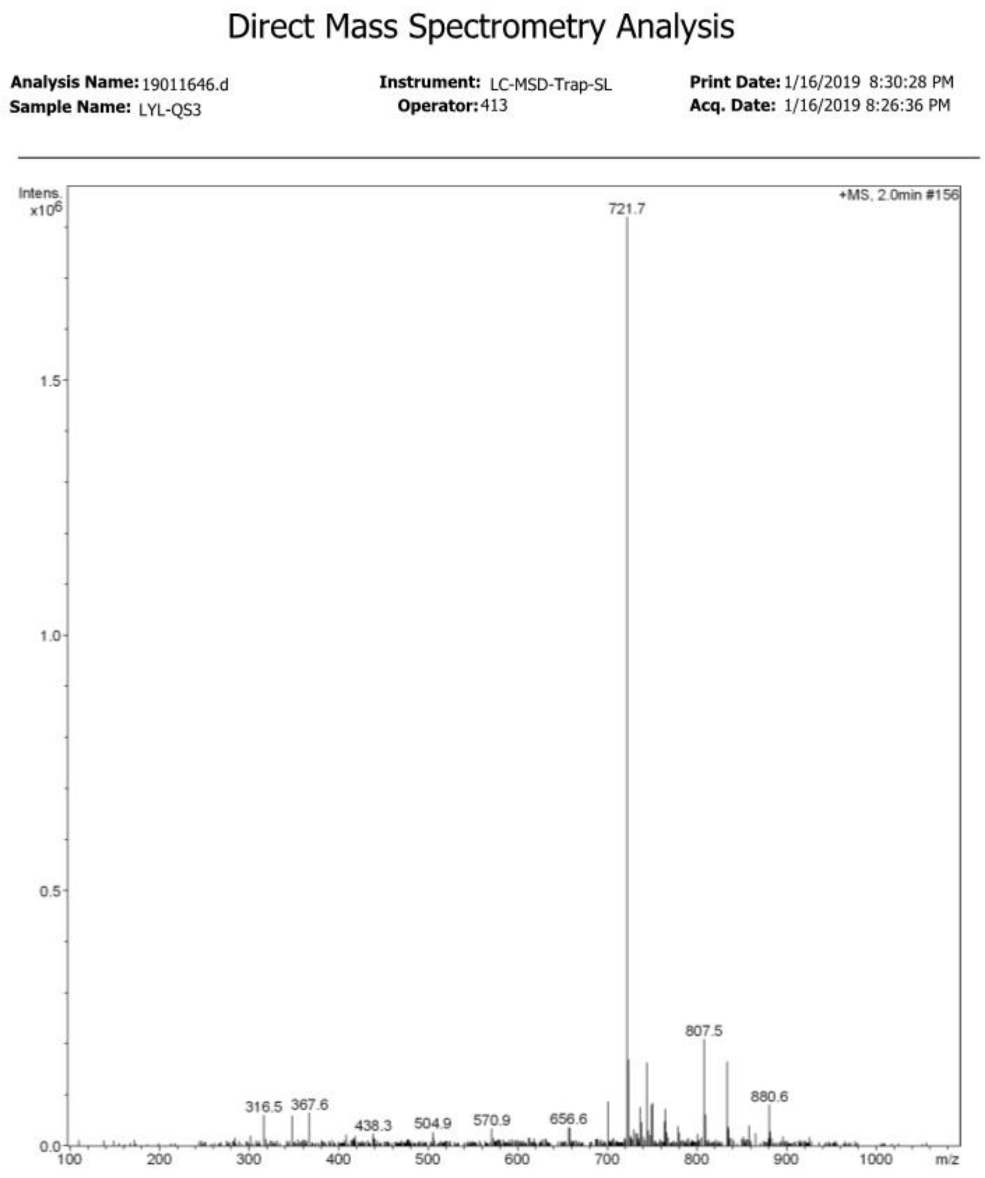

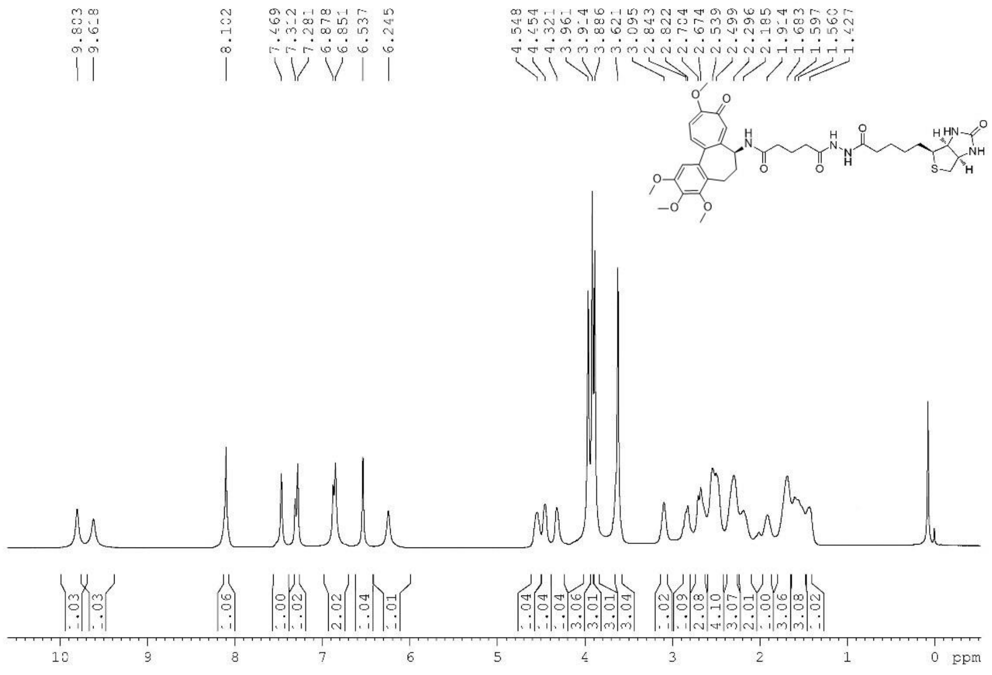

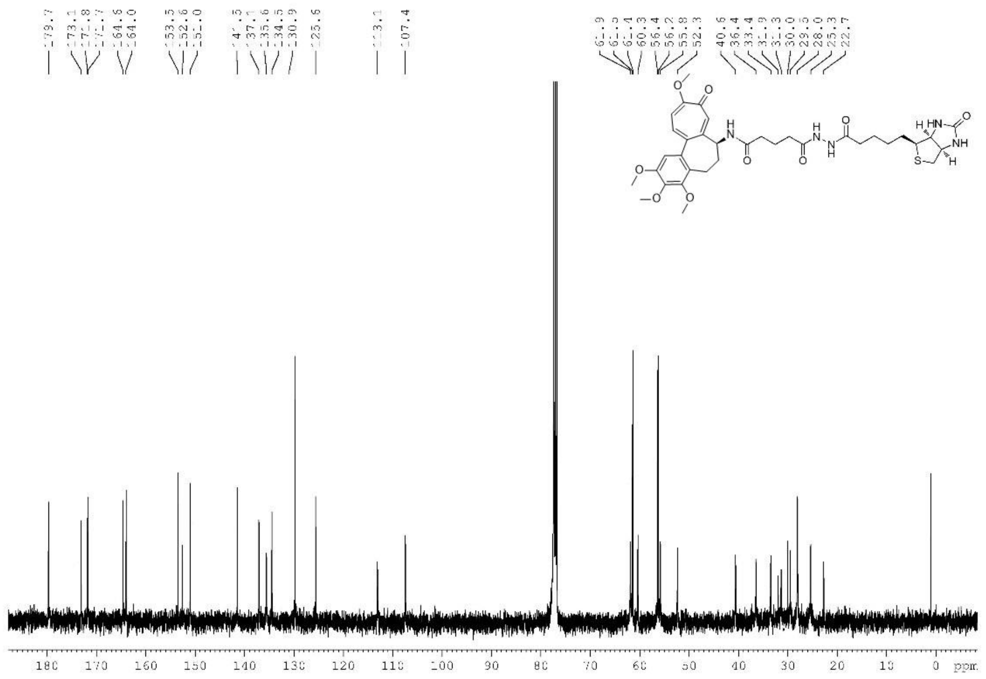
180 170 160 150 140 130 120 110 100 90 80 70 60 50 40 30 20 10 0 ppm

*2-((2-oxo-2-(2-(5-((3aS,4S,6aR)-2-oxohexahydro-1H-thieno[3,4-d]imidazol-4-yl)pentanoyl)hydrazinyl)ethyl)thio)-N-((S)-2,3,4,10-tetramethoxy-9-oxo-5,6,7,9-tetrahydrobenzo[a]heptalen-7-yl)acetamide (**11c**)*

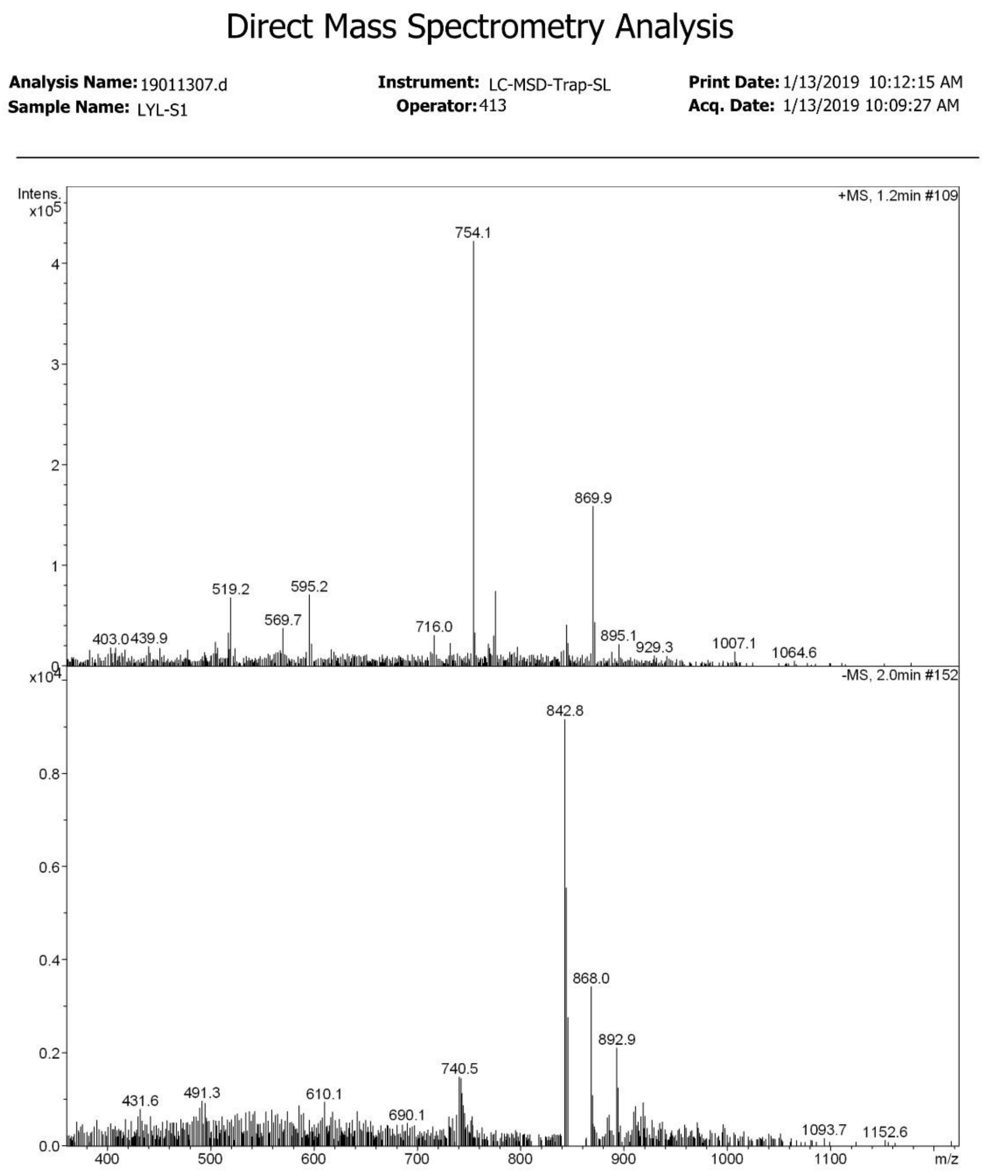

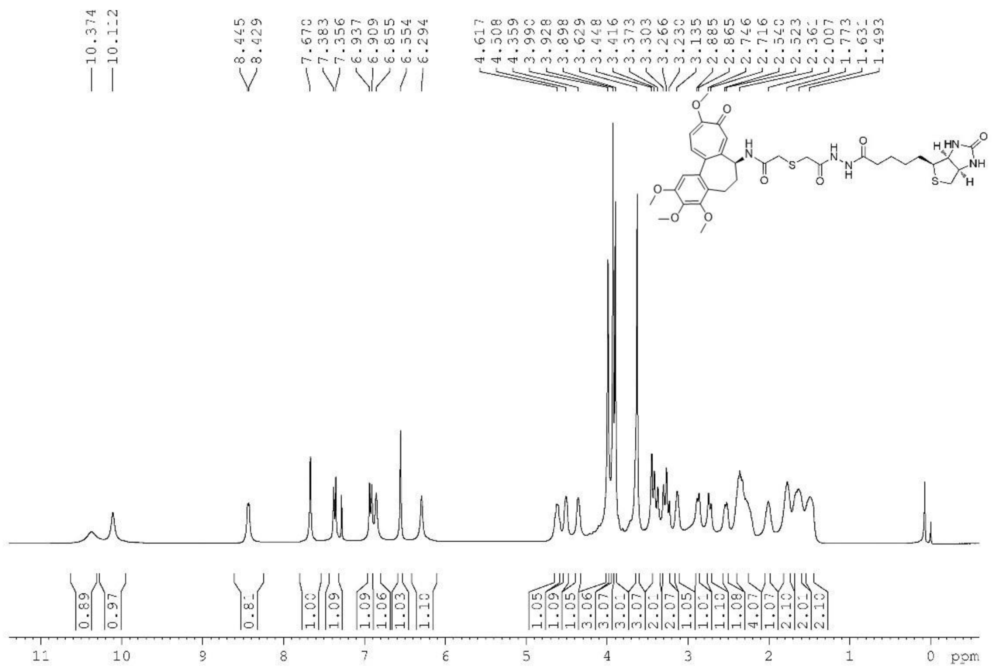

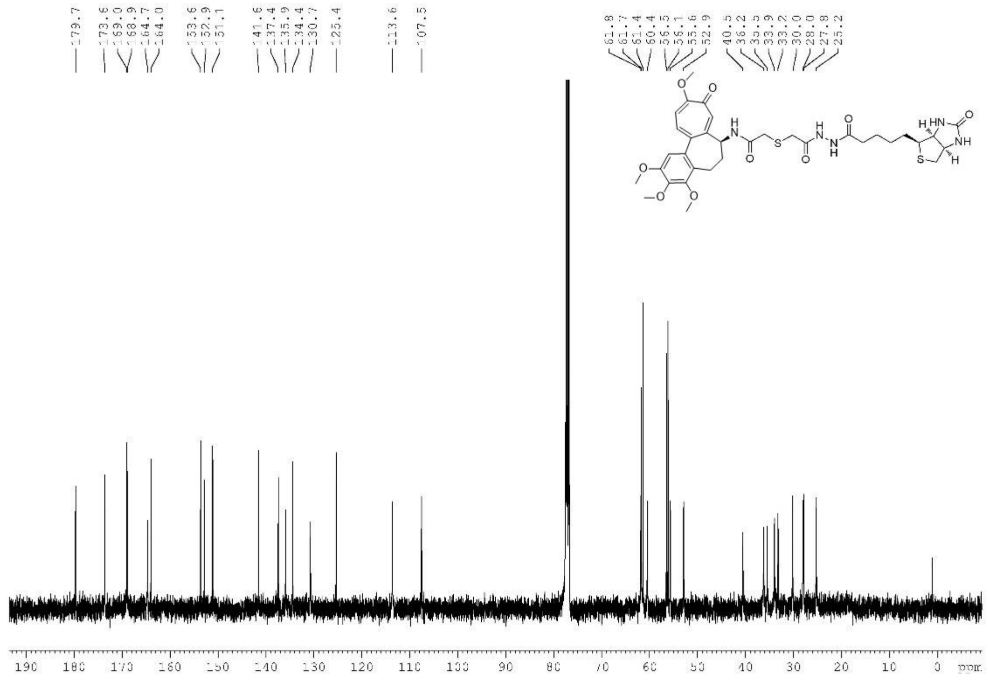

179.7
173.6
169.0
168.9
164.7
164.0
153.6
152.9
151.1
141.6
137.4
135.9
134.4
130.7
125.4
113.6
107.5
61.8
61.7
61.4
60.4
56.5
56.1
55.6
52.9
40.5
36.2
35.5
33.9
33.2
30.0
28.0
27.8
25.2
190 180 170 160 150 140 130 120 110 100 90 80 70 60 50 40 30 20 10 0 ppm
